# Disseminated Intravascular Coagulation in a High-Risk Pediatric Oncology Patient: A Pediatric Simulation Case for Residents and Fellows

**DOI:** 10.15766/mep_2374-8265.11564

**Published:** 2025-12-12

**Authors:** Kimberly Walter, Sonal Sian, Gabriela Ocampo, Jeanne Carey, Kirk Atkinson, Kaitlin Kennedy, Khawar Nawaz, Ngoc Van Horn

**Affiliations:** 1 Resident, Department of Pediatrics, University of Texas Southwestern Medical School; 2 Assistant Instructor, Department of Pediatrics, University of Texas Southwestern Medical School; 3 Instructional Design Specialist, Simulation Center, University of Texas Southwestern Medical School; 4 Clinical Operations Specialist, Simulation Center, University of Texas Southwestern Medical School; 5 Pediatric Pharmacist, Department of Pharmacy, Children's Medical Center Dallas; 6 Fellow, Department of Neonatology, University of Texas Southwestern Medical School; 7 Assistant Professor, Department of Pediatric Emergency Medicine, University of Texas Southwestern Medical School

**Keywords:** Simulation, Pediatric Hematology, Pediatric Oncology

## Abstract

**Introduction:**

Disseminated intravascular coagulation (DIC) is an acquired life-threatening condition defined by systemic coagulation imbalance. Due to the high mortality associated with this condition, it is paramount that providers quickly recognize key clinical signs and laboratory value changes.

**Methods:**

This simulation was designed for pediatric residents, fellows, or other providers who treat patients at risk for DIC. The case was a 6-year-old boy with newly diagnosed leukemia admitted to the oncology service for induction chemotherapy who presents with a nosebleed, then develops profuse, multisite bleeding consistent with a diagnosis of DIC. The goals for the team were to treat the epistaxis, verbalize DIC as the diagnosis, and address hemodynamic instability due to DIC. Directly following completion of the scenario, a debriefing session was facilitated using the PEARLS (Promoting Excellence and Reflective Learning) method. A pre- and postsimulation survey was completed that consisted of participant self-assessment of their ability to achieve each of the three educational objectives, where they rated their confidence in managing epistaxis, recognizing DIC, and achieving hemodynamic stability on a 3-point scale (1 = *almost there*, 2 = *proficient*, 3 = *mastery*).

**Results:**

Of the 91 simulation participants, which included pediatric and pharmacy residents, 89 completed the survey. The median competency score significantly increased by 0.49 points (99% CI, 0.37 to 0.61) from pre- to postsimulation (*p* < .001).

**Discussion:**

This simulation serves as a learning tool for teaching the clinical and laboratory presentation of DIC, guiding management of epistaxis, and addressing hemodynamic instability.

## Educational Objectives

By the end of this activity, learners will be able to:

1. Demonstrate at least one technique to stop active epistaxis in a pediatric patient at high risk for disseminated intravascular coagulation (DIC; in addition to applying direct pressure).
2. Verbalize the diagnosis of DIC as a cause of persistent, multisite, profuse bleeding in an oncology patient with febrile neutropenia.
3. Address hemodynamic instability in a pediatric patient with acute decompensation secondary to DIC by prioritizing stabilization of the patient's airway, breathing, and circulation.

## Introduction

Disseminated intravascular coagulation (DIC) is an acquired condition characterized by a paradoxical synchronous occurrence of both thrombi formation and widespread bleeding.^1^ This is a life-threatening condition in children, with an estimated mortality rate of 50% for overt DIC associated with shock or sepsis.^2^ It is estimated to occur in 0.4% to 1.0% of hospitalized pediatric patients.^3^ Due to the high incidence of mortality associated with this condition, rapid recognition and treatment of DIC is critical to improve outcomes. The purpose of this simulation is to improve the identification of DIC in a high-risk patient and initiate prompt treatment escalation to address bleeding complications such as epistaxis as well as hemodynamic instability.

Simulation-based training is a valuable tool for education of learners in a controlled, low-stakes environment. Simulation has been shown to enhance retention of knowledge, teamwork skills, and critical thinking.^4,5^ It also allows learners to be immersed in a life-like patient scenario without jeopardizing patient safety. Participants are given immediate feedback via a debriefing session, which reinforces educational objectives, addresses mistakes, and promotes self- and team reflection.^4^ This has been shown to improve learner clinical skills, patient safety, and clinical outcomes while also enhancing overall learner engagement.^5^

While some learners will take care of patients with DIC during their training, others may never experience this pathology in person due to the relatively low incidence of pediatric DIC. Therefore, this simulation helps to bridge this potential learning gap between clinical experiences of various learners and also better prepares trainees for this life-threatening situation. The modality of simulation was chosen for teaching this topic to enhance the learner's immersion into this topic. The physical exam findings (including multisite bleeding and even bleeding from the conjunctiva), rapid vital sign changes, and rapid decompensation are critical aspects of DIC pathology that can be appreciated by an immersive, interactive experience with a high-fidelity mannequin, digital technology, and a facilitator-controlled vitals monitor. This promotes more active engagement and a sense of realism that contributes to learner investment in the learning experience and forces them to think quickly on their feet. This type of immersive experience would not be possible in a team-based learning environment, lecture hall, or case study review. Furthermore, management of DIC requires interdisciplinary communication for rapid escalation of care. The approach involves residents, hematology and oncology providers, nurses, pharmacists, blood bank team members, respiratory therapists, and the intensive care unit team. Simulation-based training is the ideal approach to practice interdisciplinary and team communication in a safe environment.

There are limited peer-reviewed sources for teaching DIC in a pediatric patient using simulation. A search of *MedEdPORTAL* for the term DIC revealed one simulation case from 2014,^6^ discussing a case of shock leading to DIC in a 44-year-old patient. A search for the term pediatric DIC identified one simulation case from 2021,^7^ which focused on recognition of hyperleukocytosis and treatment of potential complications such as hyperkalemia due to tumor lysis syndrome, DIC, and neurologic changes. Objectives for this case did not include management of epistaxis or bleeding leading to airway compromise. A broader search did not reveal any additional educational simulation materials relevant to this subject, indicating a need for medical trainees.

This simulation curriculum aims to help learners identify the diagnosis of DIC and initiate management. Maintaining a high clinical suspicion of DIC in high-risk patients and rapidly recognizing the clinical and laboratory signs of DIC is of utmost importance to appropriately escalate care, treat the underlying cause, address hemorrhage and/or thrombosis, and decrease mortality.

## Methods

### Development

We developed this simulation scenario with a multidisciplinary team as a part of our Foundations of Simulation pediatric residency elective. We based the scenario on a real clinical case observed by the author and adapted for educational value. The faculty simulation director, a pediatric emergency medicine physician and an instructional design specialist, supervised the simulation development, editing, and execution. The simulation clinical operations specialists and the simulation pharmacy supervisor vetted the scenario and three pediatric colleagues peer-reviewed the scenario. Appendix A includes all scenario authors. The University of Texas Southwestern Medical School Institutional Review Board reviewed this activity and determined it to be nonregulated research (case number STU20250705).

The audience of this simulation are pediatric residents, fellows, or other providers who may encounter patients at high risk of developing DIC and/or severe epistaxis with airway compromise. There was no preevent material for participants to read. We executed this simulation from October to November of 2024 during a longitudinal simulation curriculum for pediatric residents. There were four total simulation event days with the scenario running four times per day (for a total of 16 occurrences). The participants included approximately 100 medical residents and eight pharmacy residents. Co-debriefing was performed with the pharmacy facilitators.

### Equipment

The sessions occurred in a patient room at a simulation center, meant to mimic an inpatient oncology room. We provided all materials and equipment necessary for completing this simulation in the simulation room. We reviewed a list of medications to be available for use during this simulation with the pharmacy team. Appendix B provides a complete list of the required materials, equipment, and medications. We utilized a high-fidelity male mannequin, Laerdal Pedi HAL, that had microphone and intubation capability, which allowed the facilitator to speak for the patient. A custom AI-generated avatar of the patient's bedside nurse was projected onto a TV screen in the room to further prompt learners. Laboratory, imaging, and physical exam findings were also projected on this screen (Appendix C).

### Personnel

Team size during the simulation sessions ranged from five to eight medical residents and one pharmacy resident. We limited active participants in the simulation to six medical residents per session, with the remainder of the residents participating in an observer role. The residents in the observer roles remained active participants in the prebriefing and debriefing sessions. The limitation in number of actively participating residents per session was aimed to avoid overcrowding and encourage input from less experienced or timid learners. This was also due to outside factors of having limited time and space in the simulation center. Each session included one medical facilitator and one pharmacy facilitator, both trained in debriefing via the PEARLS (Promoting Excellence and Reflective Learning) method. One trained SIM operations specialist managed the equipment, monitors, and mannequin during these sessions.

### Implementation

Before participants arrived, the facilitator checked that all equipment and supplies were working correctly and accessible in the room (Appendix B). Prior to the start of the simulation, the facilitator hosted a 10-minute prebriefing session to review the basic assumption, the fiction contract, and confidentiality principle, and to establish a safe environment and orient the team to the case (Appendix D). After prebriefing, a brief introduction to the case (Appendix A) was read to the participants. The participants then moved to the simulation room for a 15-minute simulation. Once the scenario started, the facilitator utilized the simulation script (Appendix A). Laboratory values and imaging findings (Appendix C) were displayed at appropriate times after being requested by participants. If additional prompting was needed via the nurse avatar, the facilitator utilized these prompts (Appendix A). After completion of the scenario, a 25-minute debriefing was held (Appendix E) and educational materials reviewed. After completion of the debrief, an optional survey was handed out to all participants (Appendix F).

### Debriefing

We utilized the PEARLS debriefing method for these simulation sessions and a debriefing guide was created (Appendix E) to assist facilitators. The debrief started with an introduction to discuss the purpose of the debrief, reinforce the basic assumption and confidentiality, and obtain learner consent to start feedback. The debriefer then assessed participants’ initial reactions and perception of the case. Next, the debriefer performed a formal discussion regarding facilitator observations of the anticipated educational objectives using the Advocacy-Inquiry method, or PAAIL (Preview, Advocacy 1, Advocacy 2, Inquiry, Listen), as seen on the outline. Key take-home points and educational objectives were reviewed with the team during the analysis phase and reinforced during the summary phase.

### Assessment

During the simulation, the assessment was completed by facilitators observing the scenario from a control room. To evaluate whether each educational objective was achieved throughout the sessions, facilitators completed a checklist of critical action items (Appendix A). The checklist highlights the three different critical actions (correlating with the three educational objectives) that a team must achieve to move on to the next frame in the scenario. If the team does not meet the critical action, additional prompting would be given with a trigger that aimed to direct the team to that objective. If the team still did not perform the critical action, there was an option to end the scenario and move on to the debriefing. Feedback relevant to achievement of specific educational objectives (based on items found in this checklist) was given to learners during the formal debriefing.

After debriefing was completed, we asked the participants to complete a brief evaluation survey (Appendix F). Participants provided information on their level of training and if they were in a specialty residency program. No other participant identifiers were asked in the survey. The goal of this survey is to assess the learner's perceived knowledge of the established educational objectives both before and after the simulation. We outlined details of each objective in the scoring rubric along with descriptions of different levels of proficiency, rated as 1 = *almost there*, 2 = *proficient*, and 3 = *mastery*. We developed this survey scale based on the residency Entrustable Professional Activities (EPA) ranking of learner competencies. A ranking of pre-entrustable is equivalent to the *almost there* category, entrustable is equivalent to the *proficient* category, and exceeding expectations is equivalent to the *mastery* category. The wording of these categories was chosen to be more familiar and simplistic, which enables the participants to quickly self-evaluate their skills. Two pediatric attendings and two pediatric residents reviewed and discussed this survey scale prior to survey implementation.

Based on Kirkpatrick's Four Levels of Training Evaluation,^8^ the survey's level of evaluation could be scored as Level 2: learning, as it assesses the degree to which participants acquire the intended confidence in their skills and knowledge after their participation in the educational event. This level of assessment measures the quality of the educational intervention and helps determine if it improved learners’ confidence in their knowledge and skills required to achieve the objectives.

## Results

Facilitators monitored the performance of each group of medical and pharmacy residents during the simulation. Throughout this simulation, all 16 learner groups that completed the scenario were able to provide at least two methods for management of the initial epistaxis. All groups were able to verbalize the diagnosis of DIC, though several required triggers (ie, prompts seen in the critical action list). All groups addressed the patient's desaturations by performing suctioning and placing the patient on oxygen. Many groups ordered blood products and escalated the patient's antibiotics. Several groups had adequate time to perform successful intubation. None of the groups had to end the scenario early secondary to failing to perform a critical action.

Surveys were voluntarily completed by residents after completion of the debriefing session. The survey was completed by 89 of 91 residents who participated in the simulation between October and November 2024. Of the 89 completed responses, 54 of the residents were categorical pediatric residents, five were internal medicine pediatric residents, eight were pediatric neurology residents, six were triple-board (pediatrics-psychiatry-child psychiatry) residents, three were pediatric pharmacy residents, and 13 did not answer. Of these residents, 37 were PGY 1, 32 PGY 2, 18 PGY 3, and two PGY 4. Physician facilitators included the primary author and a chief resident. A pediatric pharmacist also co-facilitated the groups and provided valuable input during debriefing sessions.

On pre- and postsimulation surveys using the 3-point scale, participants rated their self-assessed confidence in their ability to manage epistaxis, recognize DIC, and triage airway, breathing, and circulation. Results showed a statistically significant increase in the median competency score from before to after the simulation, increasing by 0.49 points (99% CI, 0.37 to 0.61; *p* < .001 by paired *t* test). The Figure shows the median scores from the 89 survey responders from pre- and postsimulation surveys broken down for each of the three educational objectives.

**Figure.**
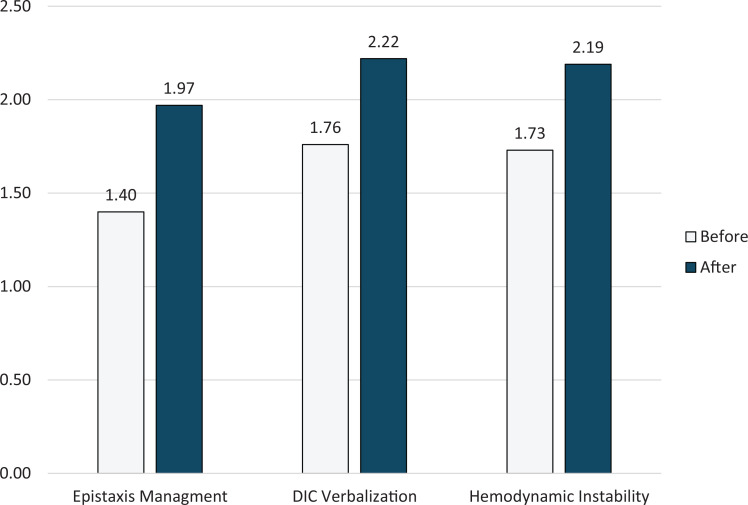
Median presimulation and postsimulation survey responses for the three educational objectives rated on a 3-point scale (1 = *almost there*, 2 = *proficient*, 3 = *mastery*). Abbreviation: DIC, disseminated intravascular coagulation.

We further stratified the analysis by postgraduate year to assess changes in perceived competency across training levels (Table). We performed Wilcoxon signed-rank tests to compare paired pre- and postsimulation median scores for each objective. Significant improvements in self-assessed competency were observed for all three objectives among PGY 1 and PGY 2 residents (all *p* < .01). PGY 3 residents showed significant improvement for educational objectives 2 and 3 (each *p* < .001) but not for educational objective 1. PGY 4 data was limited and changes in median scores did not reach statistical significance.

**Table.**
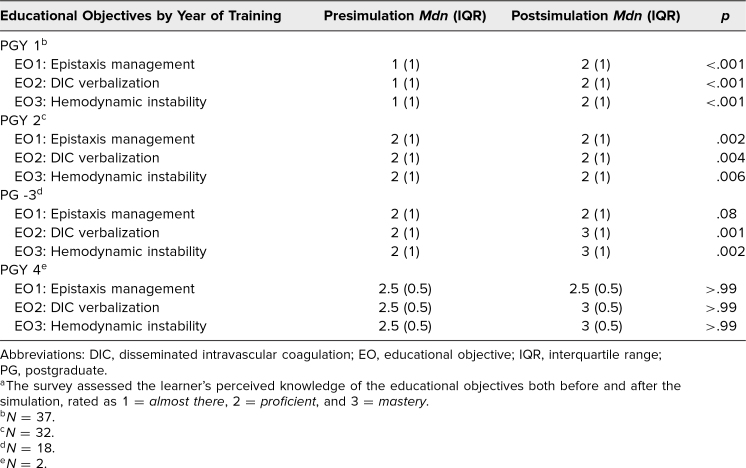
Resident Self-Assessed Pre/Postsimulation Competency by Year of Training^a^

## Discussion

The purpose of this simulation is to provide a safe learning environment for improving recognition of DIC, treatment of epistaxis, and management of hemodynamic instability. The results showed a statistically significant increase in median competency score for each of the three educational objectives from pre- to postsimulation, suggesting that learner's confidence in their knowledge and skill level regarding the three educational goals was improved after completing the training. This change was consistent despite the level of training.

Across all residents, the median self-assessed competency scores improved significantly for each of the three objectives (*p* < .001). When further broken down into year of training, it appears the most significant impact of this simulation in terms of pre- and postsimulation median survey scores was with the PGY 1 group (*p* < .001). The median score of competency most commonly increased from 1 (*almost there*) to 2 (*proficient*). For participants at training levels PGY 2 and PGY 3, the presimulation median competency score was in the *proficient* category, which is likely reflected by the additional year(s) of training and exposure to these clinical objectives. For PGY-3 participants, the pre- and postsimulation median competency scores increased from 2 (*proficient*) to 3 (*mastery*) for educational objectives 2 and 3, though not for educational objective 1. Based on this result, more dedicated time for debriefing will be required to address objective 1 in future simulations.

Overall, there was a statistically significant improvement in self-assessed competency for residents of all levels of training in achieving the stated educational objectives. While not all residents achieved a level of *mastery*, especially from PGY-1 learners, the simulation still showed improvement in self-assessed proficiency across the board. These results support this simulation method as an effective teaching tool for improving learner confidence in recognition and management of epistaxis, DIC, and resulting hemodynamic instability.

This simulation provides a simulated case of DIC recognition and management novel to the literature, involving a pediatric oncology patient with a course complicated by severe hemorrhage and airway/hemodynamic compromise. While there have been other simulation cases describing DIC,^6,7^ this case is unique in that the patient is a pediatric oncology patient (as opposed to an adult septic patient^6^) in an in-person simulation environment (as opposed to virtual^7^), utilizing real-time changes in vital signs, physical exam findings, and facilitator feedback. This simulation-based training creates an immersive, low-stakes environment for learners to improve their critical thinking skills and work in a multidisciplinary team to address real-time changes in a patient's condition and achieve the stated educational objectives.^4,5^ Furthermore, since DIC is a relatively rare, but deadly condition in the pediatric clinical care setting,^3^ this simulation helps to bridge the gap between clinical experiences of various learners. This simulation is especially beneficial to learners who may not see DIC during their training, as it creates a realistic immersive experience that forces learners to think, act, and react quickly, similar to a real-life scenario.

Limitations to this simulation include that it was only tested on pediatric and pharmacy residents and only performed at one site within one residency program. The simulation center available for this simulation had several high-fidelity mannequins and simulation technology, which is not available at every educational site or in every residency program. This simulation might also be performed with a low-fidelity mannequin in a setting with fewer resources. The facilitator would need to verbalize different physical exam changes and vital signs, such as “the patient is having hematemesis” or “the oxygen saturations have dropped to 80%.” Moulage could still be utilized to depict multisite bleeding in this case.

This simulation utilized several media images portraying different clinical scenarios (bleeding from eyes, vomiting blood). In this simulation, it was difficult to accurately portray active epistaxis or bleeding from the eyes on a mannequin. Therefore, residents often would have to ask, “Is the patient still bleeding?”; this could have potentially contributed to a delay in treatment or diagnosis in this case.

We recognize that there are limitations to a retrospective pre/postsimulation survey, especially as they were both given concurrently and after the intervention. While it may have been preferable to give a presimulation survey prior to the residents starting the simulation, this would have primed the learners to the topic of the upcoming scenario and therefore tainted the learning experience. However, by giving the presimulation survey after the educational session, it also may have allowed residents to more accurately self-evaluate their prescenario knowledge of these topics. Furthermore, the survey scale we used for self-evaluation was developed as a simplified version of the EPA competency rubric to be more user-friendly and was not an officially validated survey scale.

In conclusion, this simulation scenario is designed to assist residents, fellows, and other health care providers in recognizing DIC and managing complications including epistaxis and hemodynamic instability. The simulation method was chosen to create an immersive experience for learners, as it has been shown to improve learner clinical skills and patient safety in a safe environment.^4,5^ Because DIC has such a high (∼50%) mortality rate, detection of DIC is essential for initiation of timely intervention, management of complications, and improvement of patient outcomes.^2,3^ This scenario was well received by participants and resulted in increased confidence in their skills and knowledge regarding the three educational objectives.

## Appendices


DIC Case and Critical Action List.docxEnvironmental Preparation.docxLabs, Imaging, Prompts, Handoff.pptxPrebriefing Materials.docxDebriefing Materials.docxEvaluation Form.docx
*All appendices are peer reviewed as integral parts of the Original Publication.*
