# Supplementary material for: Disseminated Intravascular Coagulation in a High-Risk Pediatric Oncology Patient: A Pediatric Simulation Case for Residents and Fellows
Source: MedEdPORTAL. 2025 Dec 12;21:11564. doi: 10.15766/mep_2374-8265.11564 (PMC12698868; doi:10.15766/mep_2374-8265.11564)
Supplement: Supplementary file 1 — DIC Case and Critical Action List.docxEnvironmental Preparation.docxLabs, Imaging, Prompts, Handoff.pptxPrebriefing Materials.docxDebriefing Materials.docxEvaluation Form.docx [file mep_2374-8265.11564-s001.zip › A. DIC Case and Critical Action List.docx]

| **Appendix A: DIC Case and Critical Action List**    **SIMULATION CASE TITLE:** Disseminated Intravascular Coagulation (DIC) in a High-Risk Pediatric Oncology Patient: A Pediatric Simulation Case for Residents and Fellows    **AUTHORS:** Kimberly Walter, MD, Sonal Sian, MD, Gabriela Ocampo, MD, Jeanne Carey, M. Ed, RN, Kirk Atkinson, Kaitlin Kennedy, RPH, Khawar Nawaz, MD, and Ngoc Van Horn, MD    **LEARNER AUDIENCE:** Pediatric Residents, fellows | |
| --- | --- |
| **PATIENT NAME:** Alfie Wren    **PATIENT AGE:** 6    **CHIEF COMPLAINT:** Nosebleed    **PHYSICAL SETTING:** Inpatient Oncology Floor | |
|  | |
| **Brief Narrative Description of Case** | Patient is a 6-year-old male with newly diagnosed B-ALL who is currently admitted to the oncology service for induction chemotherapy and is being treated for febrile neutropenia. The patient has 1 PIV and an IVAD (Implanted Venous Access Device) site. The scenario begins with the bedside nurse calling the provider to bedside for epistaxis that has been unresponsive to 10-minutes of direct pressure. In this scenario, the epistaxis will not respond to first- or second-line treatments. Labs will show thrombocytopenia, anemia, neutropenia, and coagulopathy (fibrinogen, PTT, PT/INR, and d-dimer values will be consistent with DIC diagnosis). The patient will develop bleeding from several sites, including his IVAD and other mucosal sites (mouth, nose, eyes). Monitors will show hypotension and tachycardia. Once the diagnosis of DIC is suspected, blood products should be ordered, oncology consulted, and the PICU called. Before the PICU arrives, the patient will have profuse vomiting with desaturations and gasping/shortness of breath. Bedside suctioning should be utilized and an airway cart should be readily available to prepare for intubation. RSI medications should be discussed with pharmacy. Prior to intubation, the PICU team or anesthesia will arrive and the simulation will end. If time permits, the simulator may allow for an intubation attempt by the team per his or her discretion. The simulation is aimed at residents, fellows, and other team members who answer rapid response or code pages. |
| **Primary Learning Objectives** | By the end of this activity, learners will be able to:   1. Demonstrate at least two techniques for stopping active epistaxis in a pediatric patient at high risk for DIC 2. Verbalize the diagnosis of DIC (disseminated intravascular coagulation) as a cause of persistent, multi-site, profuse bleeding in an oncology patient with febrile neutropenia 3. Address hemodynamic instability in a pediatric patient with acute decompensation secondary to DIC by prioritizing stabilization of the patient’s airway, breathing, and circulation |

| **INITIAL PRESENTATION** | | | |
| --- | --- | --- | --- |
| **Initial Vital Signs** | HR: 110, sinus rhythm  BP: 110/70  RR: 16  SpO2: 97%  T: 38.5  Cap Refill: 3s | | |
| **Overall Setting and Appearance** | Setup:   - Moulage:   - Bleeding around dressing of IVAD/central access site   - Blood-soaked gown from nosebleed   - Blood-soaked gauze at bedside from prior bleeding - High Fidelity manikin with microphone and intubation capability - Equipment or supplies present on manikin at the beginning of the scenario:   - One PIV   - IVAD/central line   - Leads   - IV bag of cefepime attached to patient - Special equipment needs:   - Suction   - Crash cart, drawers taped except intubation supply drawer   - Intubation equipment   - Oxygen hook up on wall or cylinder   - Ambu bag   - Non-rebreather mask, Nasal Cannula   - Material for nasal packing   - Video screen access for Avatar, physical exam findings, and lab results   - Pre-written patient handoff and morning labs printed at bedside   - Cooler for blood products with tubing attached - Monitors:   - HR, RR, BP, Temp, SpO2 | | |
| **Standardized Participants (and Their Roles in the Room at Case Start)** | Media:   - Patient handoff and morning labs will be printed at bedside (see Appendix D) - Any necessary labs and imaging will be presented on a TV screen in the simulation room - There will be images of physical exam findings on an AI generated image of a 6-year-old patient that will also be displayed on the TV screen - Avatars of the patient’s nurse will be utilized to provide prompts during the scenario, including:   - “That is a lot of bleeding, what could we give to treat this?”   - “What do you think is going on?”   - “What else should we do?”   - “Look, he is starting to bleed from his eyes now!”   - “This patient is bleeding from everywhere! I saw some bleeding from his central access as well when we drew labs. What do you think is going on and what should we do next?”   - “He just had a huge vomit of blood! He is having a hard time talking to us and is gasping for air”   - “That oxygen level is awfully low. Should we do something about it?”   - “I am worried about his airway with all of the bleeding and now the desaturations. What should we do?” | | |
| **HPI** | Initial Information to be Shared with Learners by Facilitator: (Prior to entering room)  The patient is a 6-year-old male with newly diagnosed B-ALL admitted to the oncology floor for induction chemotherapy. Earlier today, he had a fever of 38.6C and was started on cefepime for febrile neutropenia, which is running now through the patient’s IVAD. There is no clear source of infection at this time. The nurse has just called you to bedside due to concern for a nosebleed that started approximately 10-minutes ago. The nurse has been applying direct pressure without improvement. You arrive to evaluate and manage the epistaxis. The patient handoff from the morning will be at bedside for your reference, along with the patient’s morning labs from about 12 hours ago. | | |
| **Past Medical/Surgical History** | **Medications** | **Allergies** | **Family History** |
|  | Currently on:  Induction day 3 chemotherapy  Allopurinol q8hr  Zofran q8hr PRN  Scopolamine patch q72hr  Tylenol q6hr PRN  Cefepime q8hr | None | None |
| **Physical Examination** | | | |
| **General** | Patient sitting up in bed. There is blood-soaked gauze around the bed and packed in the nose. The gown is bloody from his nosebleed. The central line site has some bleeding around it (covered by patient gown) | | |
| **HEENT** | Normocephalic, atraumatic  PERRL, EOMI, no redness or discharge  Normal TMs  **Epistaxis from bilateral nares** | | |
| **Neck** | Supple, no masses | | |
| **Lungs** | Clear to auscultation bilaterally | | |
| **Cardiovascular** | Regular rate and rhythm, no murmurs, gallops, or rubs; pulses 2+ | | |
| **Abdomen** | Soft, non-distended. No tenderness, rebound, or guarding. No masses or organomegaly | | |
| **Neurological** | Normal muscle strength and tone, no focal deficits | | |
| **Skin** | Warm, well perfused. Blood surrounding IVAD site, no rashes, petechiae, or other skin changes | | |
| **GU** | Normal male | | |
| **Psychiatric** | Patient appears anxious | | |

Critical Actions

| **Frame** | **Description** | **Vitals** | **Triggers** | **Notes & Lab** | **Checklist** |
| --- | --- | --- | --- | --- | --- |
| **1** | Patient sitting up in bed. There is blood-soaked gauze around the bed and packed in the nose. The gown is bloody from his nosebleed. The central line site has some bleeding around it (covered by patient gown). Cefepime is hung at bedside and connected to the patient.    Patient is alert and oriented. Normal WOB, CTA. Pupils equal and reactive. Pulses 2+ | HR: 110, sinus  BP: 110/70  RR: 16  SpO2: 97%  T: 38.5  Cap Refill: 3s    Wt: 30kg | Team completes exam, continues direct pressure and positioning. Reviews bedside handoff and labs.  Team should give pharmacologic agent to treat epistaxis  **Trigger: once team treats epistaxis with pharmacologic agent -> MOVE to 2** | If team fails to give medication for epistaxis -> Avatar: “That is a lot of bleeding, what could we give to treat this?”    If team consults ENT or PICU -> “what (medication) could we give to treat this?” | The team demonstrated at least one method for stopping active epistaxis in addition to applying direct pressure using the following technique:   - Tranexamic acid - Phenylephrine spray - Aminocaproic acid - Silver Nitrate - Other: ____________ |
| **2** | Patient begins bleeding from multiple sites. Image of patient bleeding from mouth and eyes will appear on the screen with Avatar nurse voice: “Look, he is starting to bleed from his eyes now!” Patient’s central access site will also have bleeding around it. Patient may verbalize “I’m bleeding from all over!” or “there’s blood in my mouth!”    Patient is alert and oriented. Normal WOB, CTA. Pupils equal and reactive. Pulses 2+ | HR: 150  BP: 90/60  RR: 16  SpO2: 94%  Cap Refill: 3s | Team should repeat assessment and recognize changes    **If differential and management plan for DIC voiced-> MOVE to 3**    **If no differential and management plan for DIC voiced, follow path 2B** | *If the team ordered labs, post results of the specific labs that were ordered (if available in Appendix D) now    If the team calls PICU/oncology or CODE, the consultant/code team will say “We are in another emergency right now and will need you to continue management in the meantime.” | - The team verbalized the diagnosis of DIC |
| **2B** | Patient continues to bleed from multiple sources, patient is increasingly worried and scared    Avatar will say: “This patient is bleeding from everywhere! I saw some bleeding from his central access as well when we drew labs. What do you think is going on and what should we do next?” | HR: 150  BP: 90/60  RR: 16  SpO2: 94%  Cap Refill: 3s | **If no differential and management plan for DIC voiced despite lifesavers -> MOVE to 3** | If the team calls PICU/oncology or CODE, the consultant/code team will say “What do you think is going on with this patient? What are you planning to do until we get there?” They may also ask: “What is the physical exam of the patient?” and/or “What labs have you ordered so far and how are you interpreting them?” | - The team verbalized the diagnosis of DIC |
| 3 | Image of patient with large amount of blood on his sheets will appear on the screen with Avatar nurse voice: “He just had a huge vomit of blood! He is having a hard time talking to us and is gasping for air”    Patient vomiting, gasping for breath/retching | HR: 150  BP: 90/60  RR: 24  SpO2: 85%  Cap Refill: 3s | Team should assess ABCs  Team should suction, place pt on oxygen, discuss airway support, and prep supplies and RSI (rapid-sequence intubation) medications    **Trigger: The team must verbalize reassessment of ABCs and prepare for an advanced airway -> MOVE to 4**  **If team does not prepare for advanced airway despite lifesavers -> MOVE to 4** | If the team does not assess the ABCs: nurse Avatar -> “Why is he having desaturations? What can we do?”  If the team fails to prepare for intubation: nurse Avatar -> “I am worried about his airway with all of the bleeding. What should we do?”  If team calls CODE/PICU: “We are in another emergency right now and will need you to continue management in the meantime.” | The team addressed hemodynamic instability and prioritized control of ABCs in the following ways:   - Performed suctioning of airway - Placed patient on oxygen - Prepped intubation supplies - Intubation - Administration of blood products - Escalation of antibiotics - Other: ____________ |
| 4 | PICU/CODE team arrives at bedside prior to intubation. If time permits, team may attempt intubation if time permits per facilitator’s discretion |  | End scenario | PICU will ask team to “handoff” patient and summarize case |  |

**Ideal Scenario Flow**

The patient is a 6-year-old male with newly diagnosed B-ALL who is currently admitted to the oncology service for induction chemotherapy and is being treated for febrile neutropenia. The patient has 1 PIV and an IVAD site. The patient’s morning labs and handoff sheet with more history are at the bedside. (Appendix D). The scenario begins with the bedside nurse calling the provider to bedside for epistaxis that has been unresponsive to 10-minutes of direct pressure. In this scenario, the learner should trial an additional pharmacologic agent to treat the epistaxis. The epistaxis will not respond to first- or second-line treatments. At this point, the patient will develop bleeding from several sites, including his IVAD and other mucosal sites (mouth, nose, eyes). Monitors will show hypotension and tachycardia. If the learners order labs, they will show thrombocytopenia, anemia, neutropenia, and coagulopathy (Appendix D). Fibrinogen, PTT, PT/INR, and d-dimer values will be consistent with DIC diagnosis.

Once the diagnosis of DIC is suspected, blood products should be ordered, oncology consulted, and the PICU (Pediatric Intensive Care Unit) called. Before the PICU arrives, the patient will have profuse vomiting with desaturations and gasping/shortness of breath. Bedside suctioning should be utilized and an airway cart should be readily available to prepare for intubation. Rapid-sequence intubation medications should be discussed and prepared with pharmacy. Prior to intubation, the PICU team or anesthesia will arrive and the simulation will end. If time permits, the simulator may allow for an intubation attempt by the team per his or her discretion. Regardless, the team is expected to provide appropriate transition of care to the PICU team and summarize the diagnosis and management plan.

**Anticipated Management Mistakes**

1. *Failure to correctly position patient: Many learners did not correctly position the patient with active epistaxis (choosing to lay them supine versus maintaining an upright position with direct pressure to the nares). We found it helpful to have the patient verbalize “gurgling” noises and decrease the oxygen saturation for learners to recognize that the patient should be placed upright*
2. *Failure to recognize the need for advanced airway preparation: Some of our learners did not immediately recognize that the patient required airway management in the setting of profuse airway bleeding. We found it helpful to allow the pulse oxygenation to continue to drop despite suctioning and oxygen to prompt discussion about preparing an advanced airway*
3. *Failure to voice differential of DIC: Some of our teams did not verbalize the diagnosis of DIC in a timely manner. We utilized the Avatar prompts to encourage verbalization of differential diagnosis and interpretation of lab values combined with clinical presentation*
