# Supplementary material for: Disseminated Intravascular Coagulation in a High-Risk Pediatric Oncology Patient: A Pediatric Simulation Case for Residents and Fellows
Source: MedEdPORTAL. 2025 Dec 12;21:11564. doi: 10.15766/mep_2374-8265.11564 (PMC12698868; doi:10.15766/mep_2374-8265.11564)
Supplement: Supplementary file 1 — DIC Case and Critical Action List.docxEnvironmental Preparation.docxLabs, Imaging, Prompts, Handoff.pptxPrebriefing Materials.docxDebriefing Materials.docxEvaluation Form.docx [file mep_2374-8265.11564-s001.zip › B. Environmental Preparation.docx]

**Appendix B: Simulation Environment Preparation**

Resources

PALS (Pediatric Advanced Life Support) reference cards

Personal Protective Equipment: gowns, gloves

**Simulated Medications** (bolded medications are more likely to be used in this simulation)

Acetaminophen

**Aminocaproic Acid**

Ampicillin-sulbactam

Atropine

**Cefepime**

Cefotaxime

Ceftazidime

Ceftriaxone

Clindamycin

Epinephrine 1:10,000

**Etomidate**

Fentanyl

Heparin

**Ketamine**

Lactated Ringers

Lorazepam

Midazolam

Morphine

Norepinephrine

Normal Saline (NS)

Ondansetron

**Oxymetazoline spray**

Ibuprofen

**Rocuronium**

**Silver Nitrate**

Sodium bicarbonate

Succinylcholine

**Tranexamic Acid**

**Vancomycin**

Blood Products:

**Fresh Frozen Plasma (FFP)**

**Cryoprecipitate**

**Packed Red Blood Cells (pRBCs)**

**Platelets**

Equipment

High Fidelity manikin (microphone and intubation capability) in hospital gown, on bed

Monitor –

Noninvasive blood pressure (NIBP)

Heart Rate (HR)

Respiratory Rate (RR)

Oxygen saturation (SpO2)

Temperature (T)

Blood Pressure cuff, Heart Rate monitor leads, oxygen saturation probe

Oxygen hook-up- on wall or cylinder

Bag-valve-mask system

Zoll and Zoll Pads

Oxygen– nasal cannula, mask - simple and/or non-rebreather

Suction device

Shoulder roll

Endotracheal tubes suitable for mannikin size

Laryngoscope, Miller and Mac blades, size suitable for mannikin size

Stethoscopes

IV tubing/blood product tubing and filters

IV pumps, pressure bags/blood product pumps

Crash cart

Additional specific equipment for this scenario:

- Material for nasal packing
- IVAD/central access site with bleeding around it
- IV cefepime connected to patient
- Moulage (bloody patient gown, blood-soaked gauze in nares and around bedside)
- Video screen access for Avatar, physical exam findings, and lab results
- Pre-written patient handoff and morning labs printed at bedside
- Cooler for blood products with tubing attached
