# Supplementary material for: Disseminated Intravascular Coagulation in a High-Risk Pediatric Oncology Patient: A Pediatric Simulation Case for Residents and Fellows
Source: MedEdPORTAL. 2025 Dec 12;21:11564. doi: 10.15766/mep_2374-8265.11564 (PMC12698868; doi:10.15766/mep_2374-8265.11564)
Supplement: Supplementary file 1 — DIC Case and Critical Action List.docxEnvironmental Preparation.docxLabs, Imaging, Prompts, Handoff.pptxPrebriefing Materials.docxDebriefing Materials.docxEvaluation Form.docx [file mep_2374-8265.11564-s001.zip › D. Prebriefing Materials.docx]

### **Appendix D: Prebriefing Materials**

### Introductions

Today we will be participating in a simulation. I would like to start with some introductions. Would you mind going around the room and saying your name, year, and career interest? My role today is to facilitate this experience and promote reflection on your actions and decision-making in this scenario.

**Goals of the SIM are to be ready to:**

- Correctly triage and manage a rapid response situation
- Work on verbalization of a differential diagnosis, decision making, management plan, and overall thought process given an evolving clinical presentation
- Work in a collaborative fashion with our pharmacy colleagues and practice effective communication within the team

**Debriefing**

We will be starting with a 10-minute pre-brief, the simulation for 15-minutes, and then a 25-minute debrief session. During the debriefing, I will ask more about your decision-making process and the thoughts that drove your actions and decisions. I may also ask about teamwork, communication, leadership, and team roles as you adapted to the situation.

**Assessment**

Our purpose here today is for learning and development. There will not be any grading or reporting about your performance today. This activity gives you a chance to practice and learn in a safe environment.

**Basic Assumption**

I believe everyone here is capable, intelligent, cares about doing their best, and wants to improve. I believe that mistakes are a part of learning and will not be punished or judged. We expect and welcome mistakes during simulation as a learning opportunity.

**Fiction Contract**

We know that there are many gaps between this simulation and the reality of patient care. Please try your best to suspend realism and immerse yourself in the situation to make the most of this learning opportunity. In this specific scenario, there will be a lot of media images, makeup, and props to try to best recreate physical exam findings. We know they are not perfect, but please try your best to “buy in” to the overall clinical presentation.

**Confidentiality**

We ask that you are mindful of confidentiality during this simulation day. The “Vegas rule” applies here: what happens in sim stays in sim. Please don’t discuss your colleague’s performance outside of the case and please do not discuss details of this case with fellow residents. We re-use these cases and would like everyone to have the same experience without any kind of advantages going into the scenario.

**Safety**

There are some additional safety considerations I want to review. In the unlikely event that something “real” happens, such as a fire alarm or a clinical emergency, one of the faculty/staff will say: “This is not a simulation.” If you hear those words that means that this is a real event and that you should respond accordingly. Additionally, in this scenario there is nothing you can do that will “kill” the patient.

**Roles**

Please organize your team into roles and assign responsibilities as you see fit. There will need to be one team leader, which should be a second- or third-year resident and preferably a third-year resident for this specific scenario. Your scope of practice will reflect the same scope of practice expected from a resident physician at your level of training. There will be no one assigned to a role that they are not trained to be (for example, a resident will not be assigned as a respiratory therapist).

**Simulation Considerations**

The simulation will take place entirely on an inpatient floor. In this scenario, you are at an academic pediatric hospital with in-house PICU, consults, anesthesia, and surgery teams. You may verbalize making consults, calling a code, etc to utilize these resources or for extra guidance during this scene.

**Virtual Embedded Simulation Performer (ESP)**

There will be a virtual ESP via Avatar: the patient’s nurse. The avatar will guide you as needed throughout the scenario, please take her concerns or questions seriously.

**Orientation to the Case**

- The crash cart and all necessary items will be present, the drawers will be taped for any materials that you will not need. All equipment will be near the bedside or in the crash cart.
- Pharmacy Table will be present, pharmacy resident should stand behind the table. All medications will be in the room and drawn in real time.
- TV screen/Prompt: If you request labs or imaging, they will appear on the screen as relevant. Please request each lab individually. Please verbally interpret all labs and images. Changing physical exam findings will also appear on the screen, you may consider designating one person to continually check the TV screen for changes.
- Please place vital sign leads on the patient, the monitors will reflect the current vitals
- The manikin will have pulses, breath sounds, and microphone capability. The control room will notify you if there are any technical difficulties with the manikin or monitors

**Questions?**

**Prompt**

The patient is a 6-year-old male with newly diagnosed B-ALL admitted to the oncology floor for induction chemotherapy. Earlier today, he had a fever of 38.6C and was started on cefepime for febrile neutropenia, which is running now through the patient’s IVAD. There is no clear source of infection at this time. The nurse has just called you to bedside due to concern for a nosebleed that started approximately 10-minutes ago. The nurse has been applying direct pressure without improvement. You arrive to evaluate and manage the epistaxis. The patient handoff from the morning will be at bedside for your reference, along with the patient’s morning labs from about 12 hours ago.
