# Supplementary material for: Disseminated Intravascular Coagulation in a High-Risk Pediatric Oncology Patient: A Pediatric Simulation Case for Residents and Fellows
Source: MedEdPORTAL. 2025 Dec 12;21:11564. doi: 10.15766/mep_2374-8265.11564 (PMC12698868; doi:10.15766/mep_2374-8265.11564)
Supplement: Supplementary file 1 — DIC Case and Critical Action List.docxEnvironmental Preparation.docxLabs, Imaging, Prompts, Handoff.pptxPrebriefing Materials.docxDebriefing Materials.docxEvaluation Form.docx [file mep_2374-8265.11564-s001.zip › E. Debriefing Materials.docx]

### **Appendix E: Debriefing Materials**

**Phase 1: Introduction**

Thank you all for your participation in our simulation. Now we are going to start our debrief process, which will take about 25-minutes. During the debrief we will talk about your feelings overall during the sim, your decision-making process and thoughts that drove your actions in this case, and finally discuss some take home points from the experience.

Before we get started I just want to repeat some of the main concepts from our pre-briefing discussion. First, we recognize this is a simulation and is different from real life. We appreciate you immersing yourself in the situation. One is the basic assumption- we believe you are all intelligent people who want to improve and mistakes are a part of that learning process. This is a safe space and we ask that our discussion and simulation topic remain confidential. There is no formal evaluation or “grade” for this simulation, but we hope that this discussion will give you some learning points to incorporate into your practice in the future. Is it ok if we discuss a few points?

**Phase 2: Reactions (Present tense)**

- Feelings: So you all just finished a challenging scenario. How are you feeling now? Could we go around the room and come up with 1-2 words that describe how you are each individually feeling?
- Facts: Just to make sure we’re on the same page, let’s talk about what was going on here. Could we have a volunteer (that was not the team leader) tell us what was happening in this scenario?

**Phase 3: Understanding (Past tense)**

*Explore (PAAIL)*

- Preview: “I’d like to talk about…”
- Advocacy: “I saw/heard…”
- Advocacy: “I think/am concerned/worried that…”
- Inquiry: “I wonder/am curious…”
- Pause for response

*Discuss + Teach*

- Compare/Contrast
- Pros/Cons
- Clinical Stories + Examples

*Generalize to Practice:* “How might you put this into practice going forward?”

PAAIL Examples with Given Learning Objectives:

1. Epistaxis management:

- I would like to discuss the team’s management of epistaxis.
- I saw that you did an excellent job of maintaining pressure when arriving to bedside. After that, I did not hear any discussion about what the next steps might be given this persistent nosebleed.
- I think in a case of this high-risk patient, it would be appropriate to think about trying an additional medication to stop the bleeding.
- I wonder what you all were thinking at that time and if anyone had similar thoughts?

2. DIC differential and management:

- I would like to talk about the differential diagnosis in this case.
- I heard some discussions about the patient’s thrombocytopenia as a potential cause of the bleeding but did not hear any other ideas.
- In this scenario, given that the patient had a newly-diagnosed leukemia, was now febrile, had abnormal coagulation studies, and was bleeding from multiple sites, I worry that there may have been something more systemic going on.
- I’m curious what you thought about this clinical presentation?

3. Triage ABCs:

- I want to discuss your thoughts towards the end of the scenario when the patient started to become hypoxic.
- I saw that the team correctly identified that the patient was desaturating and placed the patient on nasal cannula.
- I worry that with the persistent vomiting, gasping, and desaturations that the patient’s airway may have been compromised.
- I wonder if the team considered other options for airway protection in this patient?

4. Teamwork:

- I would like to talk about your interdisciplinary teamwork and communication during this simulation.
- Once the patient began to decompensate, I heard a lot of voices talking about next steps for airway protection. I noticed that no one on the team talked to the pharmacy resident about the steps.
- Given that the patient was still alert and conscious at this time, I am worried that pharmacy was not looped in on the plan for a rapid-sequence intubation.
- I am curious if anyone thought about preparing RSI medications for this patient during the chaos of the decompensation?

**Phase 4: Summary**

I’d like to wrap up the debriefing now.

Can you tell me some takeaway points you’ll incorporate into your practice in the future?

Now that we are finished with the debriefing, I would appreciate it if each of you could fill out a brief 1-minute survey to assess your learning and understanding of the objectives. Please circle the box that most applies to you.
