# Supplementary material for: Disseminated Intravascular Coagulation in a High-Risk Pediatric Oncology Patient: A Pediatric Simulation Case for Residents and Fellows
Source: MedEdPORTAL. 2025 Dec 12;21:11564. doi: 10.15766/mep_2374-8265.11564 (PMC12698868; doi:10.15766/mep_2374-8265.11564)
Supplement: Supplementary file 1 — DIC Case and Critical Action List.docxEnvironmental Preparation.docxLabs, Imaging, Prompts, Handoff.pptxPrebriefing Materials.docxDebriefing Materials.docxEvaluation Form.docx [file mep_2374-8265.11564-s001.zip › F. Evaluation Form.docx]

### Appendix F: Simulation Evaluation Form

**Residency program? (please circle one):** Medical (categorical, med-peds, triple board, peds-neuro); Pharmacy

**What year of residency are you in? (please circle one):** 1 2 3 4 Other _____

**AFTER** participating in this session, how confident are you in your ability to: *(circle the box that most applies)*

|  | Almost There | Proficient | Mastery |
| --- | --- | --- | --- |
| Outcome #1: Perform escalation of care for epistaxis | Not comfortable with pharmacologic and non-pharmacologic management of epistaxis | Can comfortably perform both pharmacologic (afrin, TXA, amicar, silver nitrate) and non-pharmacologic (direct pressure, positioning) management for epistaxis | Considers surgical option (ENT consult) after performing both pharmacologic and non-pharmacologic interventions |
| Outcome #2: Recognize DIC as a cause of persistent, multi-site, profuse bleeding in an oncology patient with febrile neutropenia | Not comfortable recognizing DIC as a differential | Verbalizes differential diagnosis (including DIC) in the case of multi-site bleeding in a high-risk patient | Identifies differential diagnosis of DIC and first steps of management including supportive care, treating the underlying cause, and ordering blood products |
| Outcome #3: Prioritize and triage airway, breathing, and circulation and prepare to emergently manage given a changing clinical scenario | Not comfortable setting up for advanced airway | Able to prepare for advance respiratory measures including setting up suction, supplies needed for intubation, and preparing RSI medications | Recognition of difficult airway and establishing contingency plans |

**BEFORE** participating in this session, how confident are you in your ability to: *(circle the box that most applies)*

|  | Almost There | Proficient | Mastery |
| --- | --- | --- | --- |
| Outcome #1: Perform escalation of care for epistaxis | Not comfortable with pharmacologic and non-pharmacologic management of epistaxis | Can comfortably perform both pharmacologic (Afrin, TXA, Amicar, silver nitrate) and non-pharmacologic (direct pressure, positioning) management for epistaxis | Considers surgical option (ENT consult) after performing both pharmacologic and non-pharmacologic interventions |
| Outcome #2: Recognize DIC as a cause of persistent, multi-site, profuse bleeding in an oncology patient with febrile neutropenia | Not comfortable recognizing DIC as a differential | Verbalizes differential diagnosis (including DIC) in the case of multi-site bleeding in a high-risk patient | Identifies differential diagnosis of DIC and first steps of management including supportive care, treating the underlying cause, and ordering blood products |
| Outcome #3: Prioritize and triage airway, breathing, and circulation and prepare to emergently manage given a changing clinical scenario | Not comfortable setting up for advanced airway | Able to prepare for advance respiratory measures including setting up suction, supplies needed for intubation, and preparing RSI medications | Recognition of difficult airway and establishing contingency plans |

How can we improve this simulation? Any additional comments? (optional)
