# Supplementary material for: Disseminated Intravascular Coagulation in a High-Risk Pediatric Oncology Patient: A Pediatric Simulation Case for Residents and Fellows
Source: MedEdPORTAL. 2025 Dec 12;21:11564. doi: 10.15766/mep_2374-8265.11564 (PMC12698868; doi:10.15766/mep_2374-8265.11564)
Supplement: Supplementary file 1 — DIC Case and Critical Action List.docxEnvironmental Preparation.docxLabs, Imaging, Prompts, Handoff.pptxPrebriefing Materials.docxDebriefing Materials.docxEvaluation Form.docx [file mep_2374-8265.11564-s001.zip › C. Labs, Imaging, Prompts, Handoff.pptx]

## Slide 1
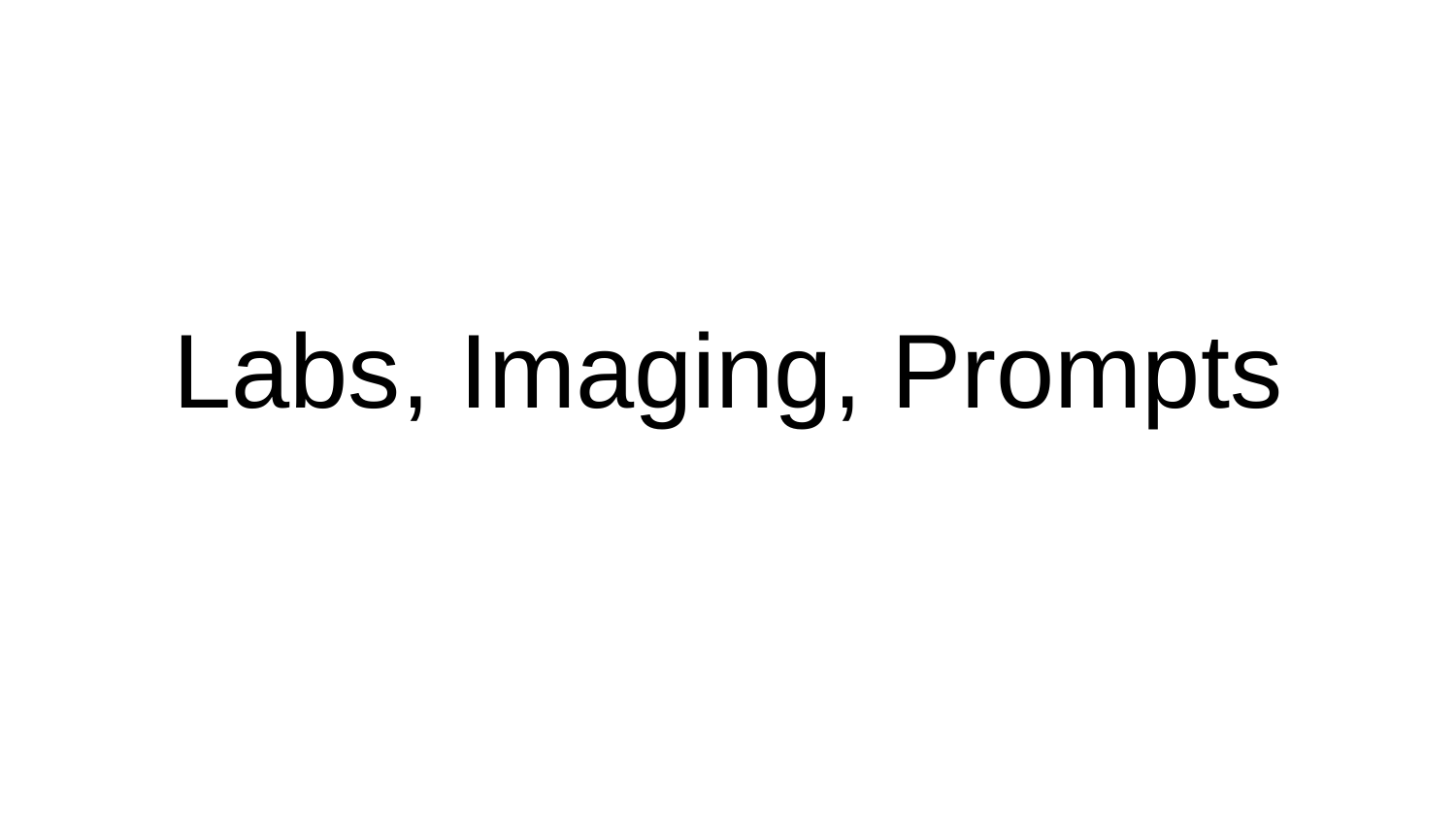

# Labs, Imaging, Prompts

## Slide 2
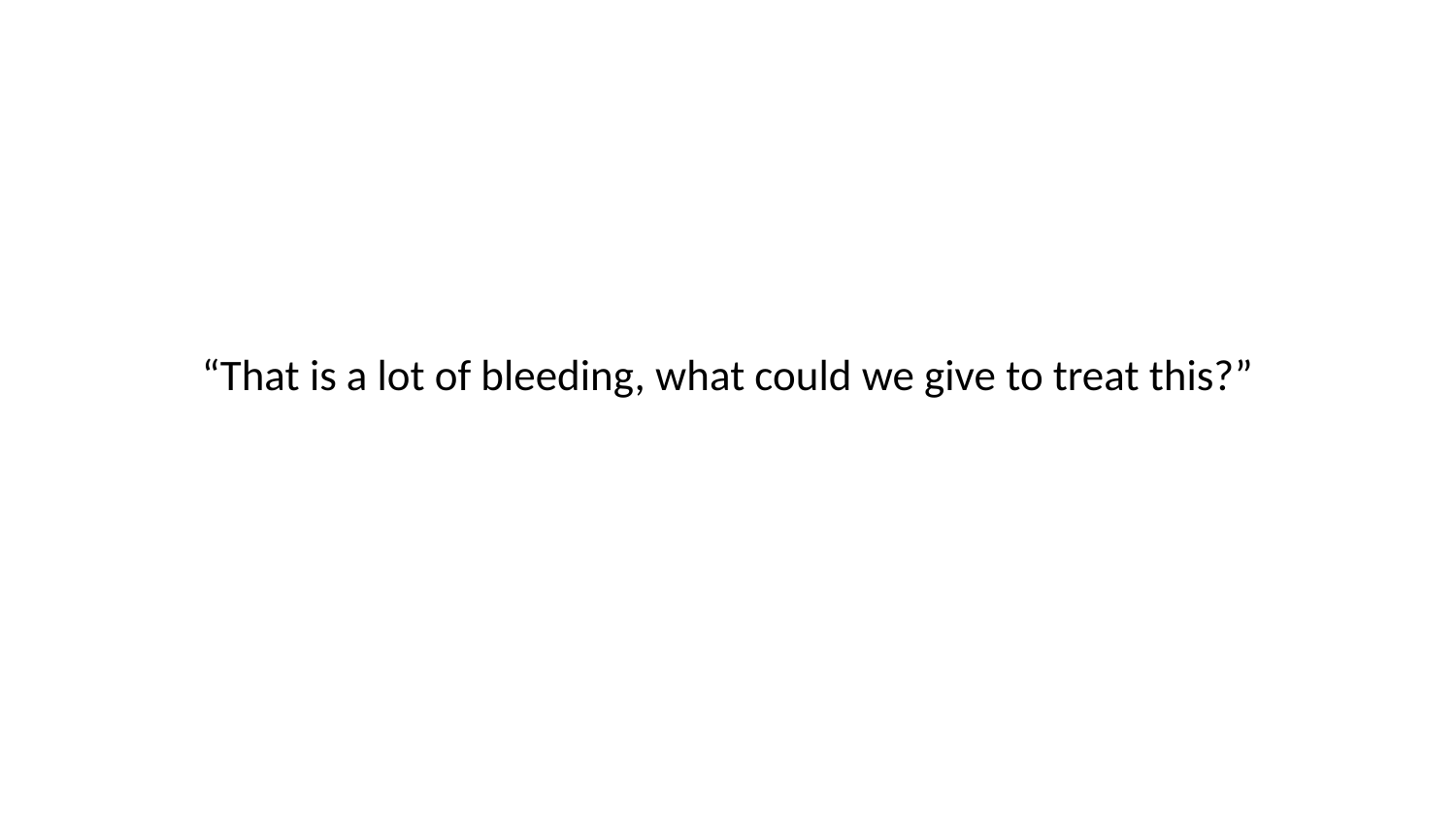

# “That is a lot of bleeding, what could we give to treat this?”

## Slide 3
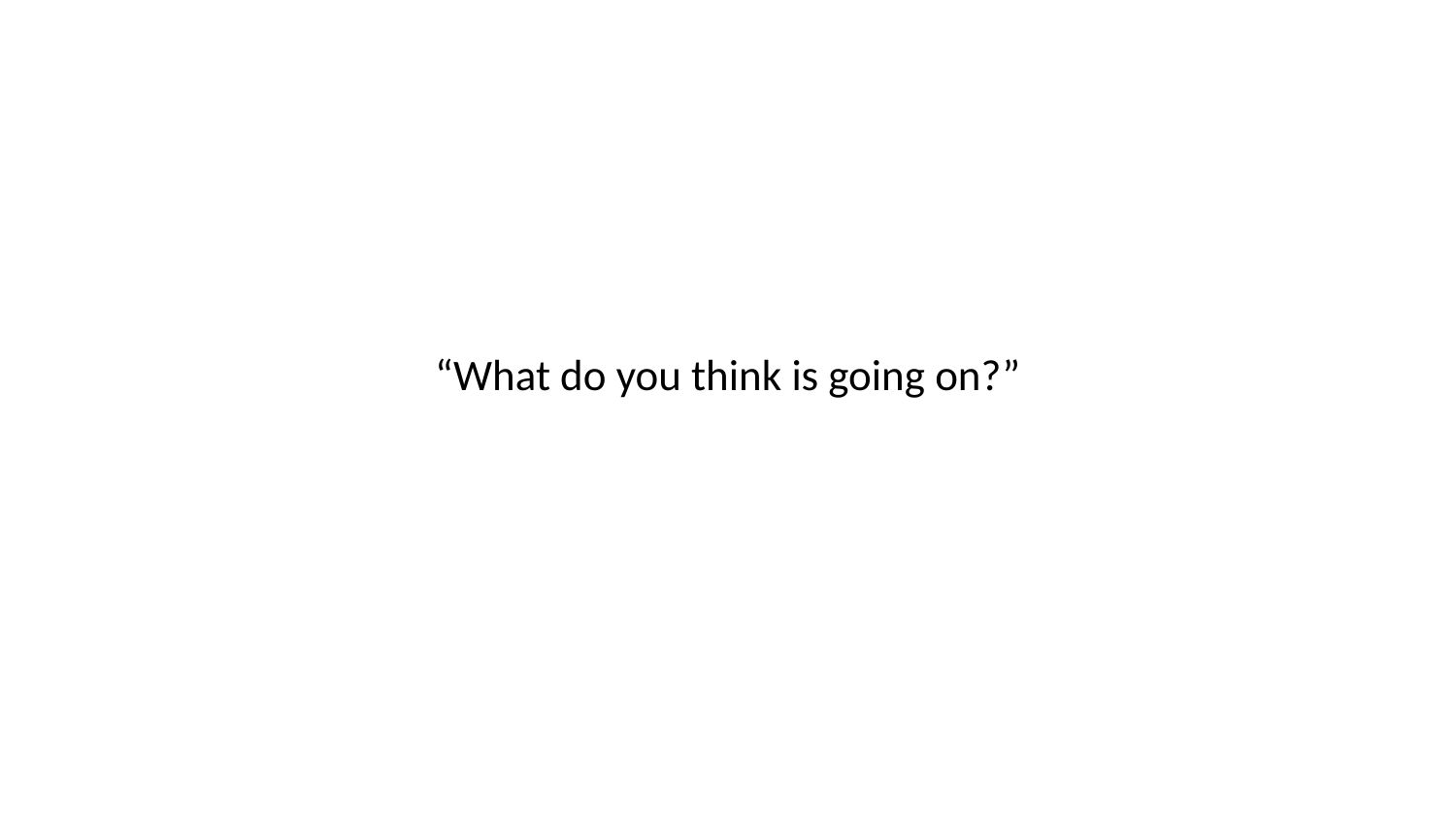

# “What do you think is going on?”

## Slide 4
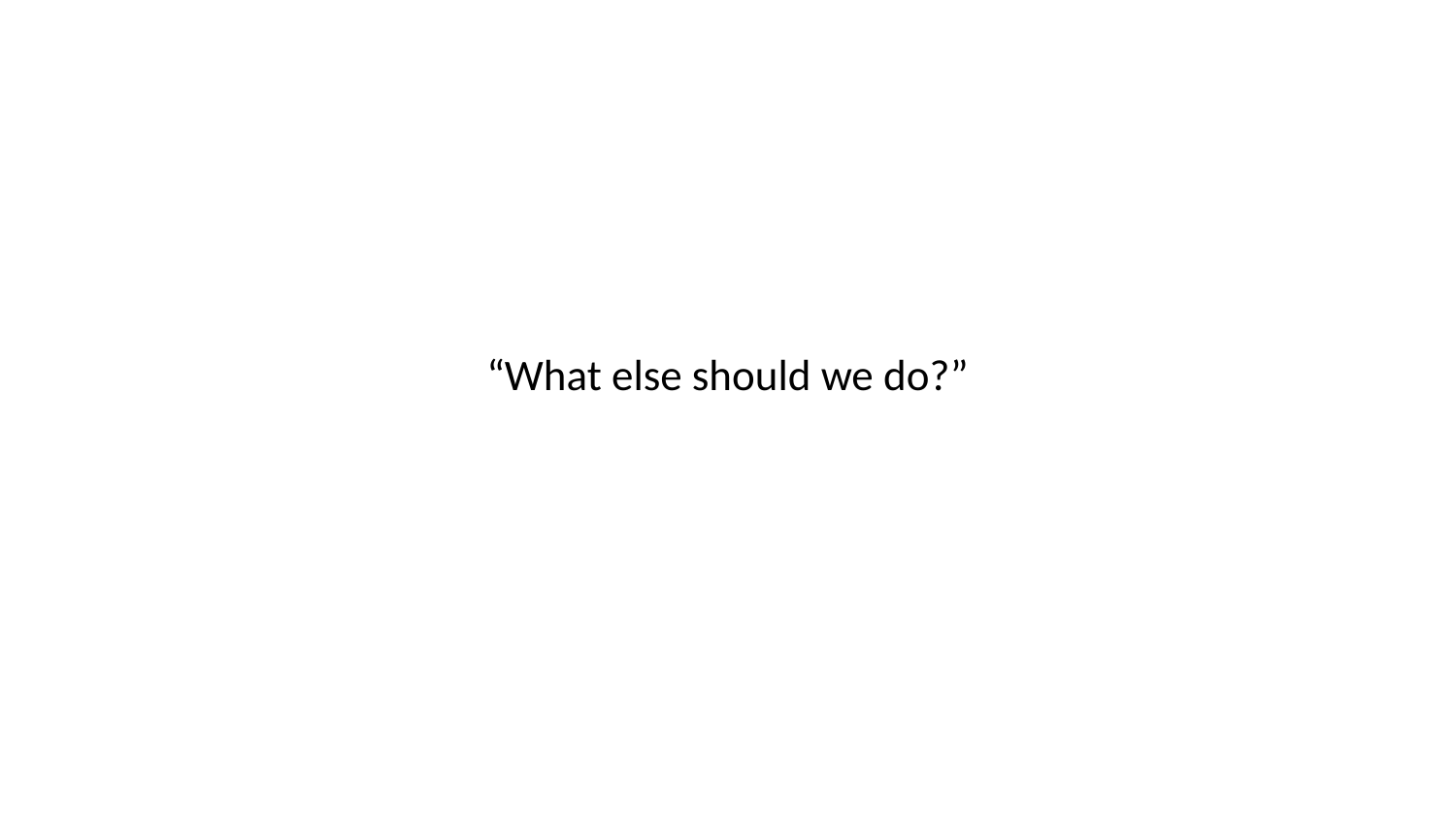

# “What else should we do?”

## Slide 5
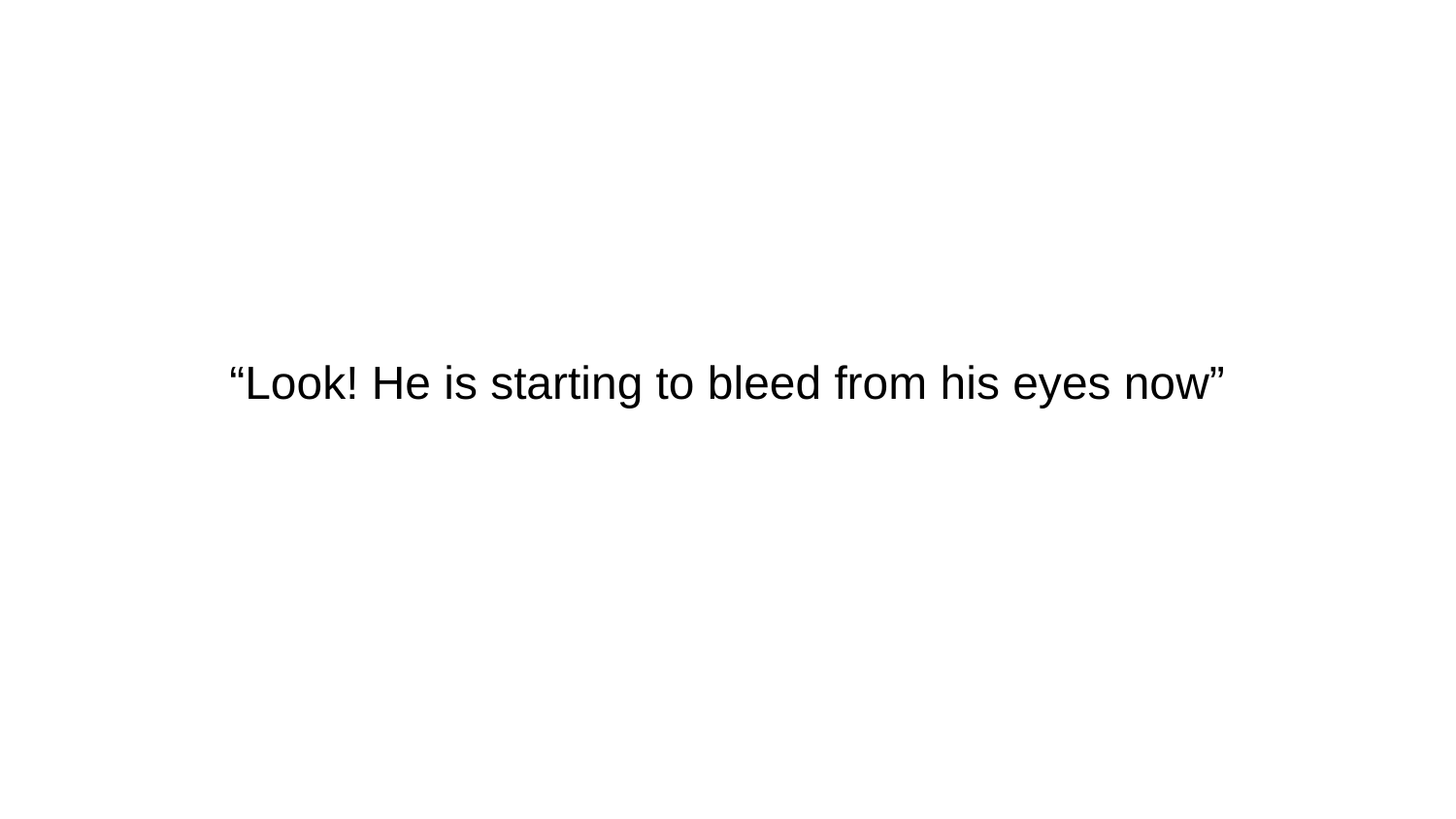

# “Look! He is starting to bleed from his eyes now”

## Slide 6
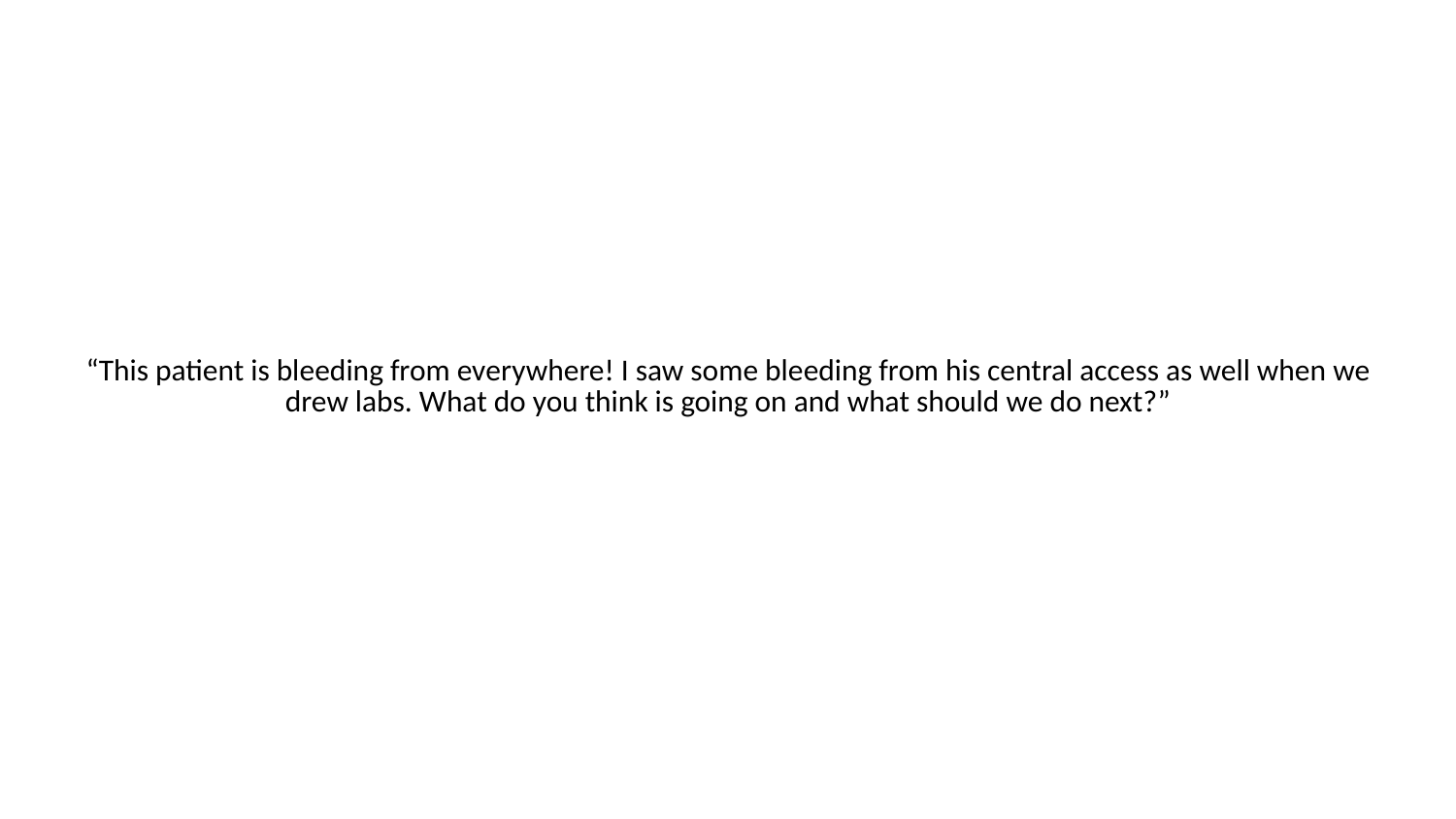

# “This patient is bleeding from everywhere! I saw some bleeding from his central access as well when we drew labs. What do you think is going on and what should we do next?”

## Slide 7
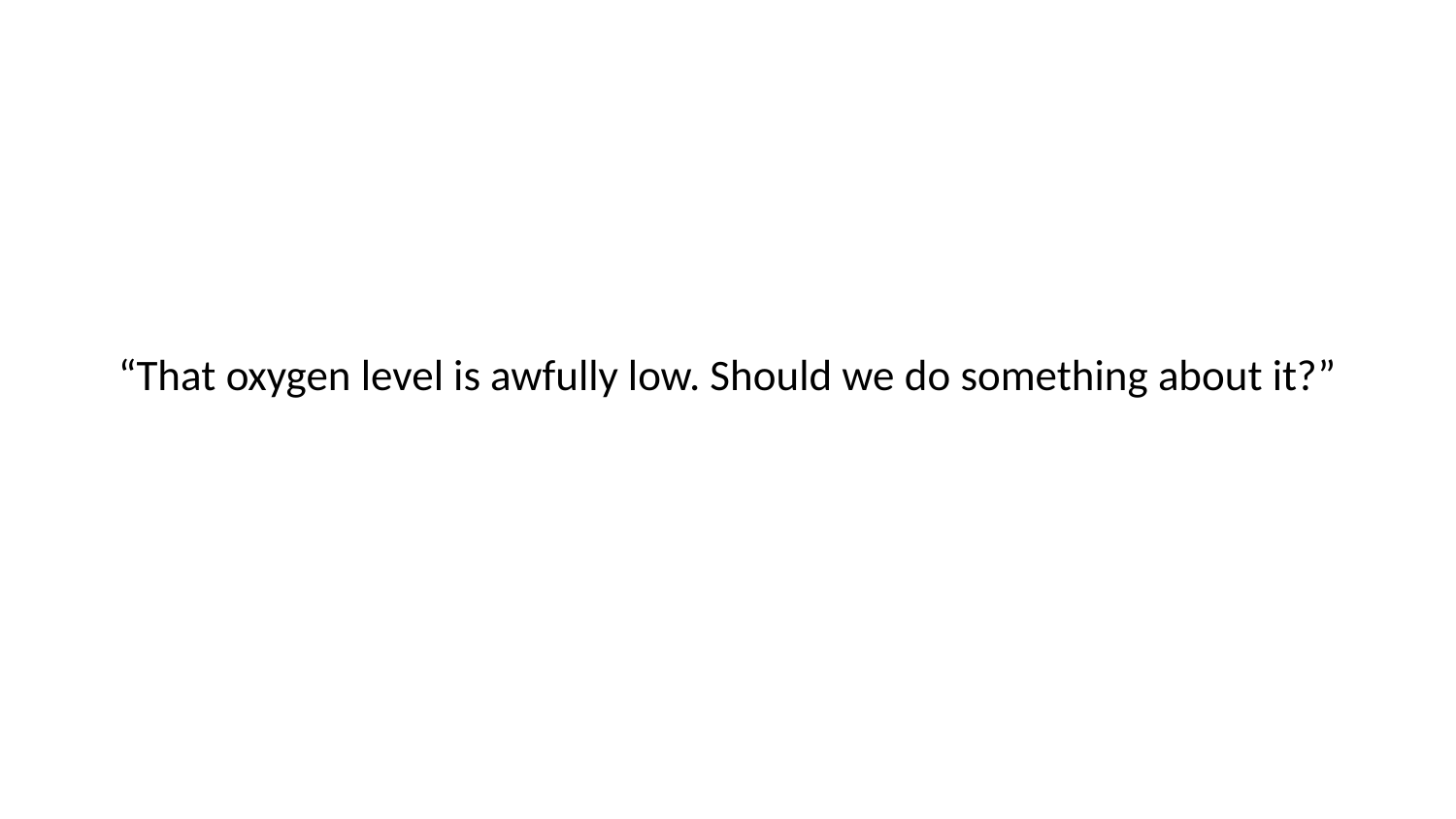

# “That oxygen level is awfully low. Should we do something about it?”

## Slide 8
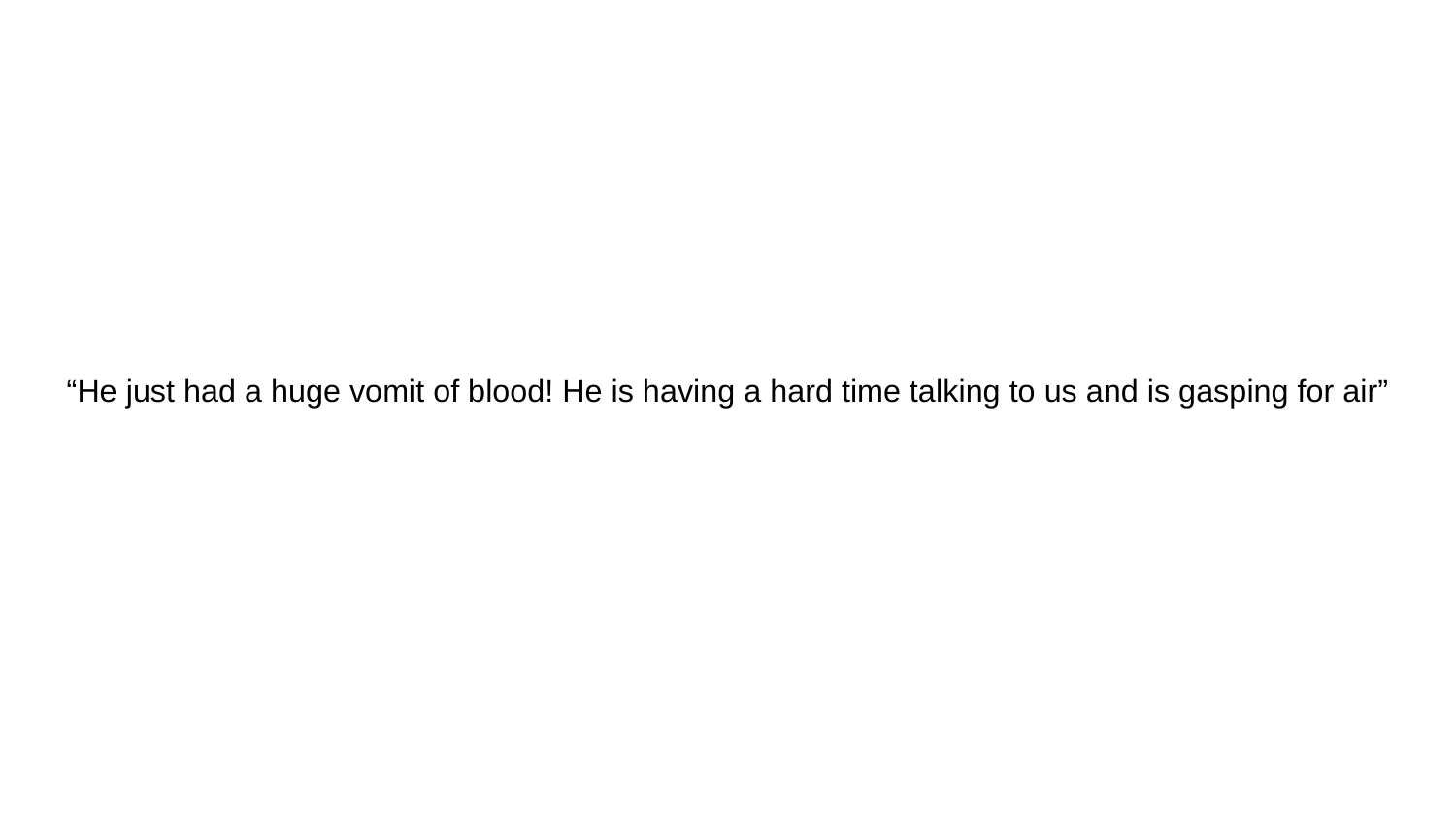

# “He just had a huge vomit of blood! He is having a hard time talking to us and is gasping for air”

## Slide 9
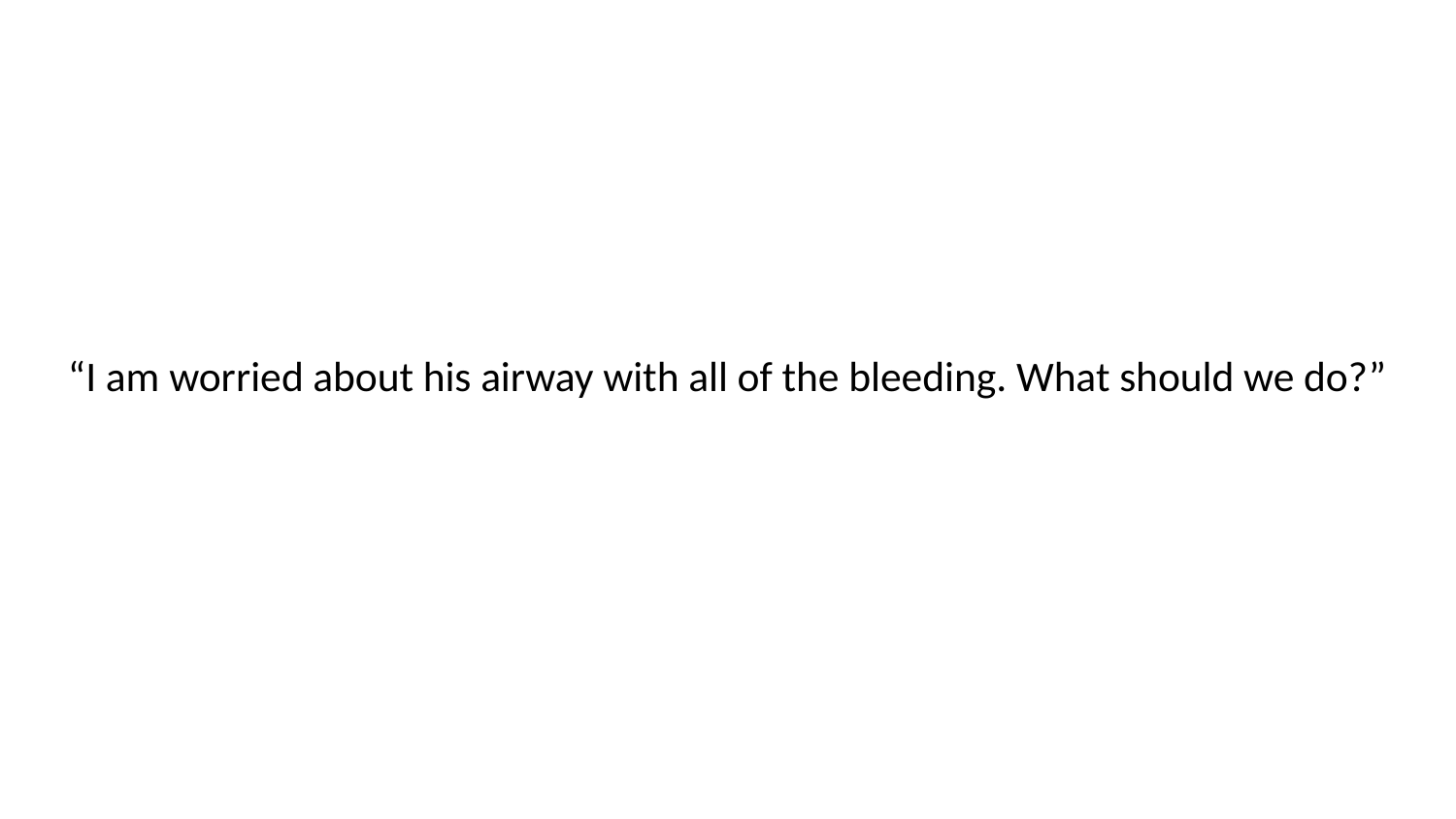

# “I am worried about his airway with all of the bleeding. What should we do?”

## Slide 10
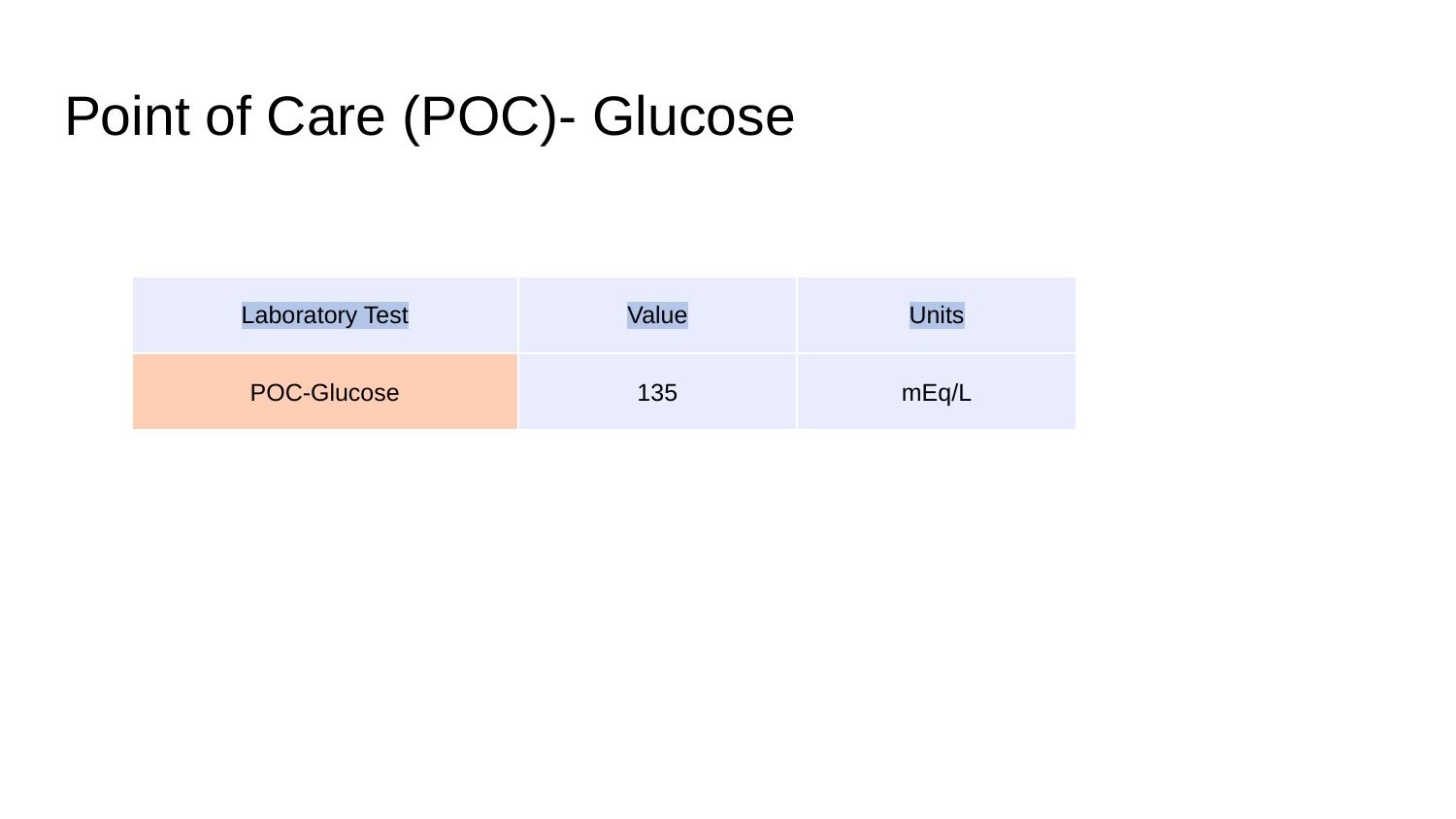

# Point of Care (POC)- Glucose
| Laboratory Test | Value | Units |
| --- | --- | --- |
| POC-Glucose | 135 | mEq/L |

## Slide 11
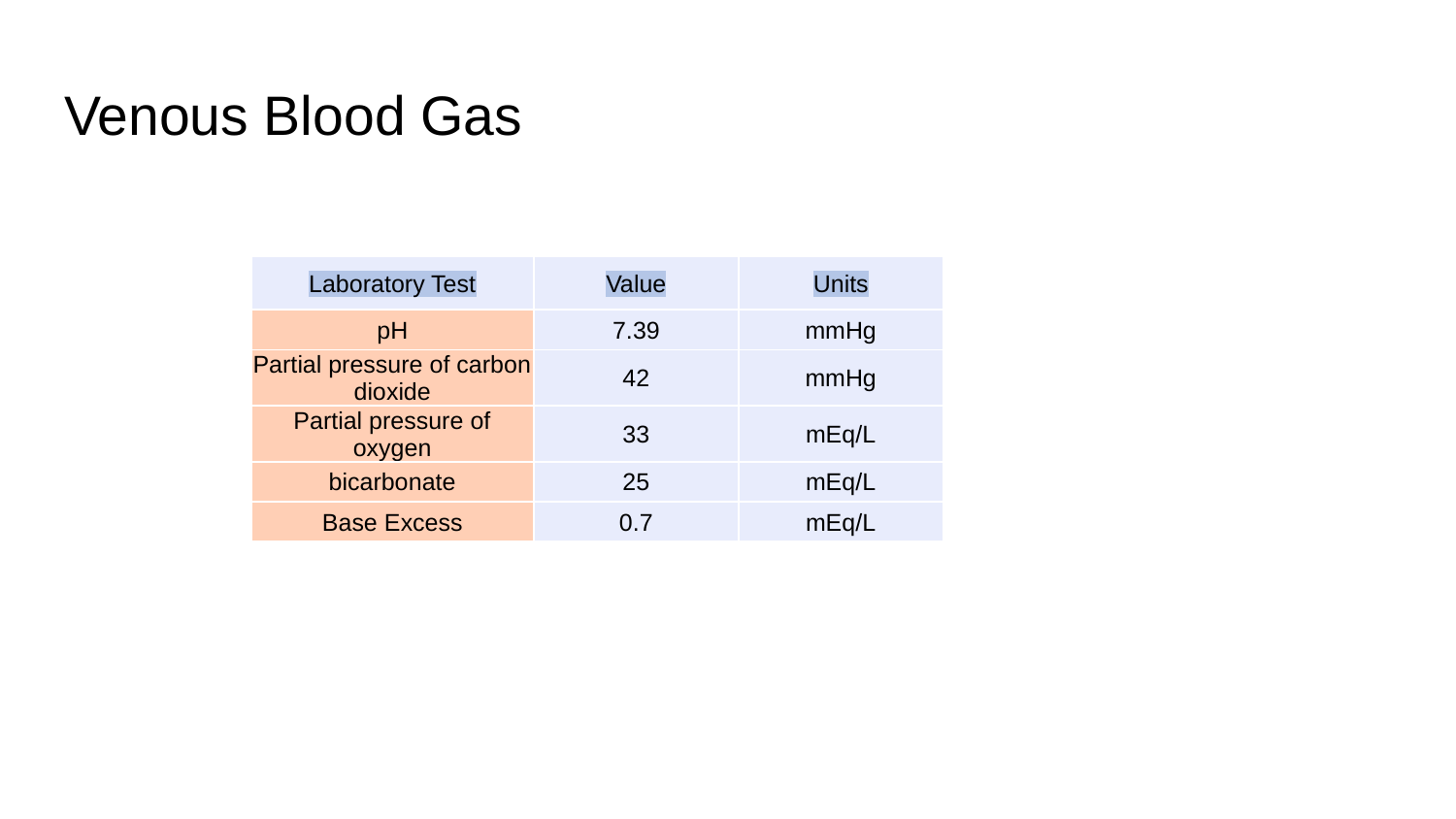

# Venous Blood Gas
| Laboratory Test | Value | Units |
| --- | --- | --- |
| pH | 7.39 | mmHg |
| Partial pressure of carbon dioxide | 42 | mmHg |
| Partial pressure of oxygen | 33 | mEq/L |
| bicarbonate | 25 | mEq/L |
| Base Excess | 0.7 | mEq/L |

## Slide 12
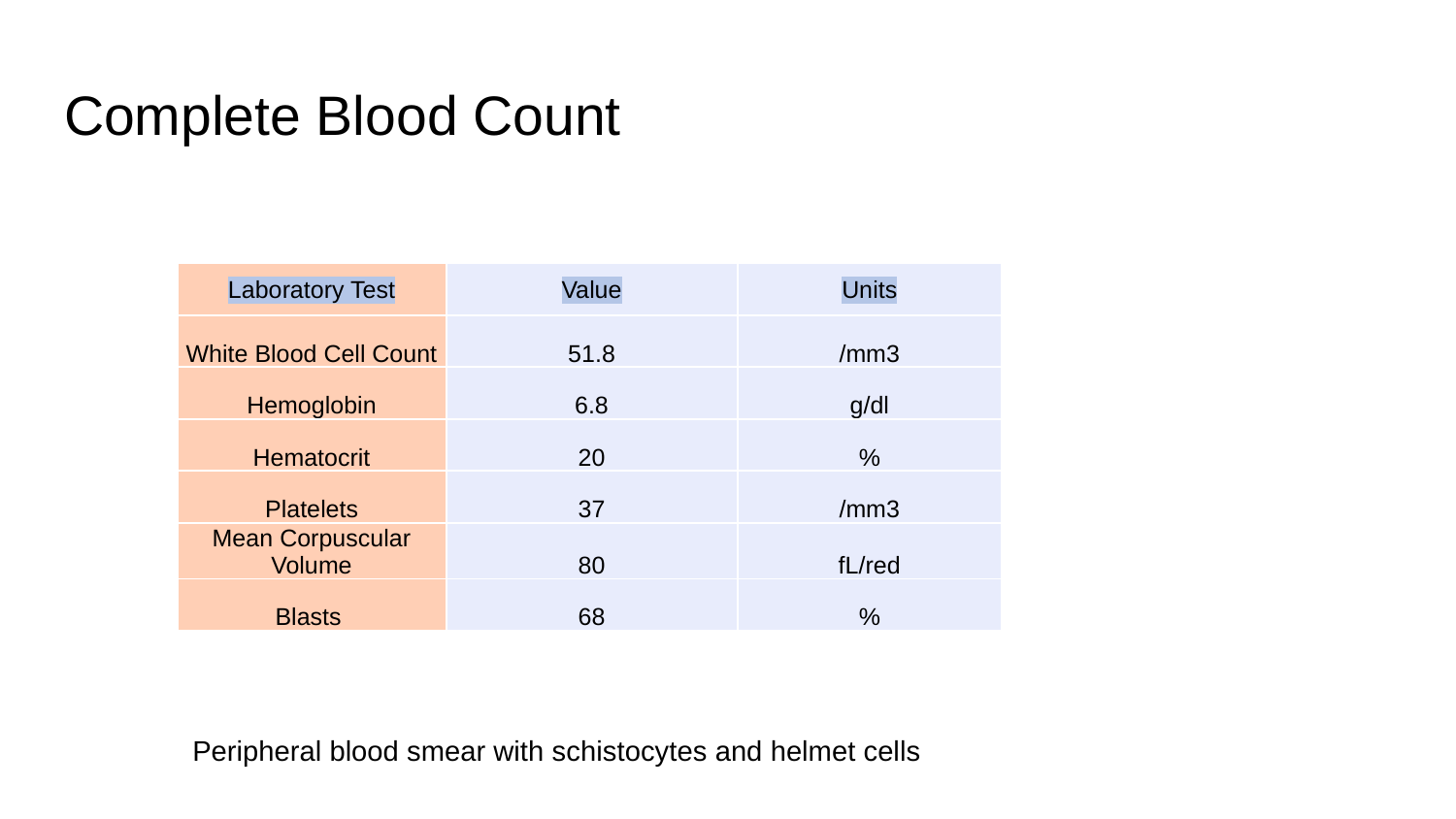

# Complete Blood Count
| Laboratory Test | Value | Units |
| --- | --- | --- |
| White Blood Cell Count | 51.8 | /mm3 |
| Hemoglobin | 6.8 | g/dl |
| Hematocrit | 20 | % |
| Platelets | 37 | /mm3 |
| Mean Corpuscular Volume | 80 | fL/red |
| Blasts | 68 | % |
Peripheral blood smear with schistocytes and helmet cells

## Slide 13
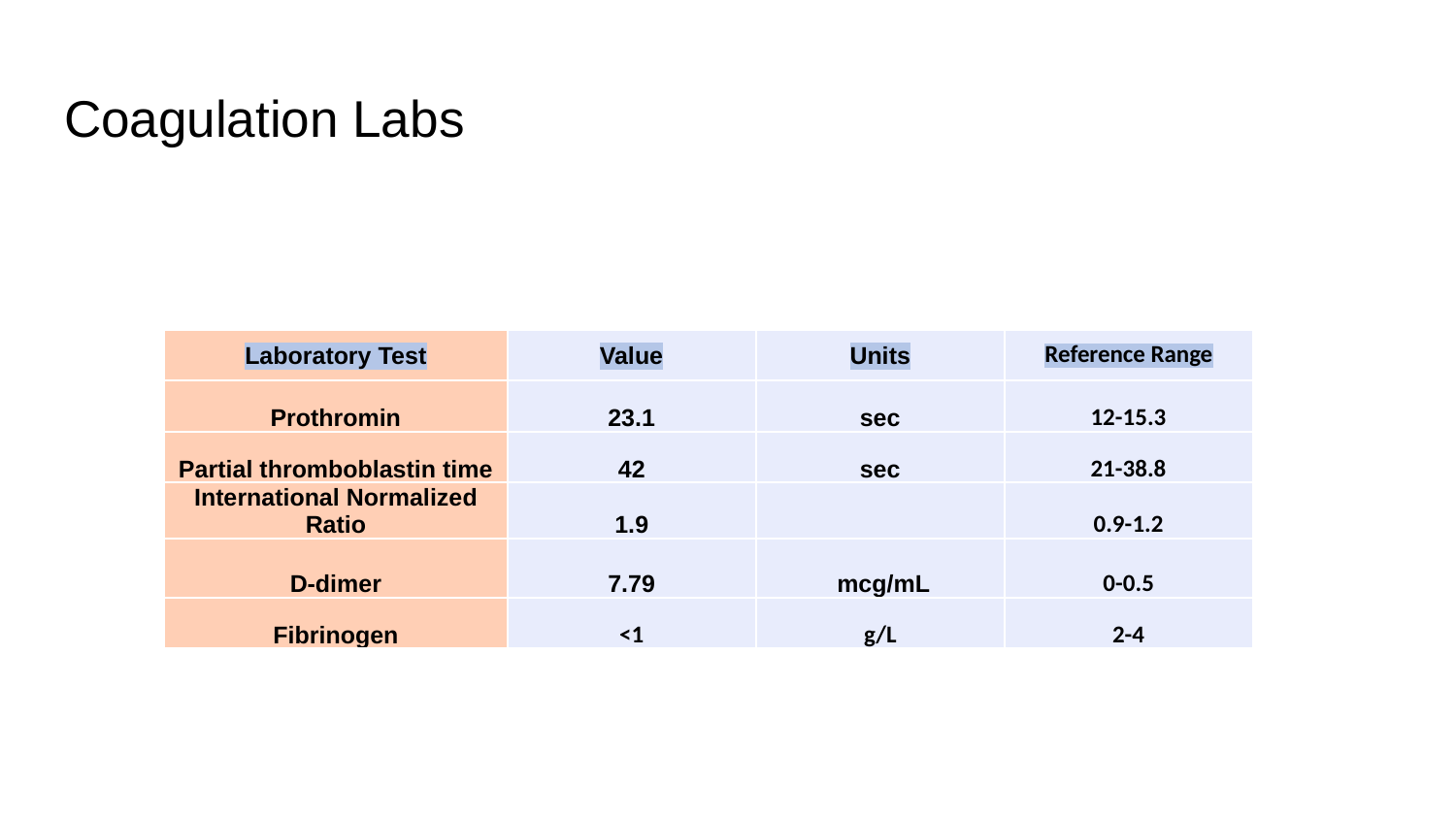

# Coagulation Labs
| Laboratory Test | Value | Units | Reference Range |
| --- | --- | --- | --- |
| Prothromin | 23.1 | sec | 12-15.3 |
| Partial thromboblastin time | 42 | sec | 21-38.8 |
| International Normalized Ratio | 1.9 | | 0.9-1.2 |
| D-dimer | 7.79 | mcg/mL | 0-0.5 |
| Fibrinogen | <1 | g/L | 2-4 |

## Slide 14
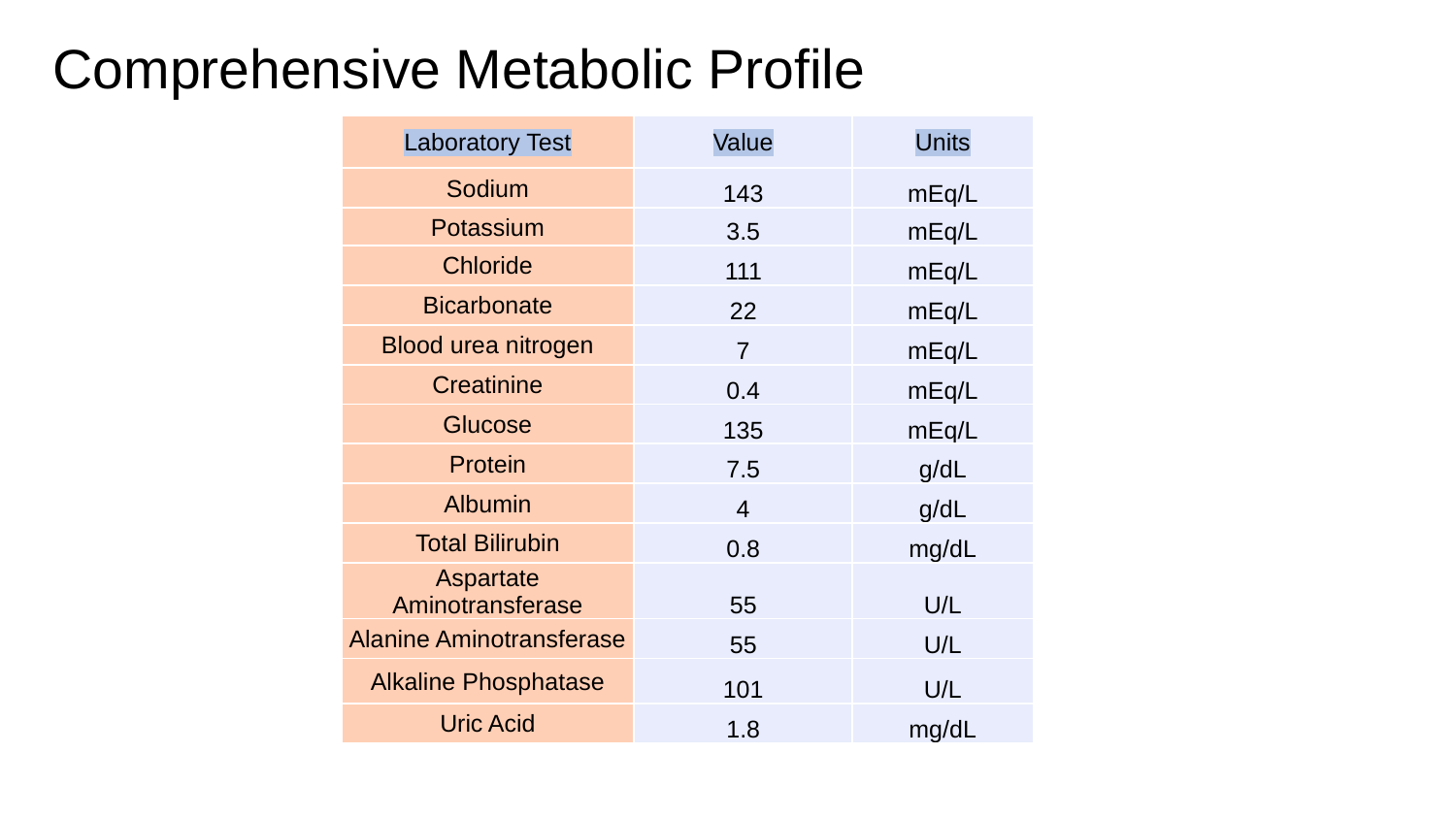

# Comprehensive Metabolic Profile
| Laboratory Test | Value | Units |
| --- | --- | --- |
| Sodium | 143 | mEq/L |
| Potassium | 3.5 | mEq/L |
| Chloride | 111 | mEq/L |
| Bicarbonate | 22 | mEq/L |
| Blood urea nitrogen | 7 | mEq/L |
| Creatinine | 0.4 | mEq/L |
| Glucose | 135 | mEq/L |
| Protein | 7.5 | g/dL |
| Albumin | 4 | g/dL |
| Total Bilirubin | 0.8 | mg/dL |
| Aspartate Aminotransferase | 55 | U/L |
| Alanine Aminotransferase | 55 | U/L |
| Alkaline Phosphatase | 101 | U/L |
| Uric Acid | 1.8 | mg/dL |

## Slide 15
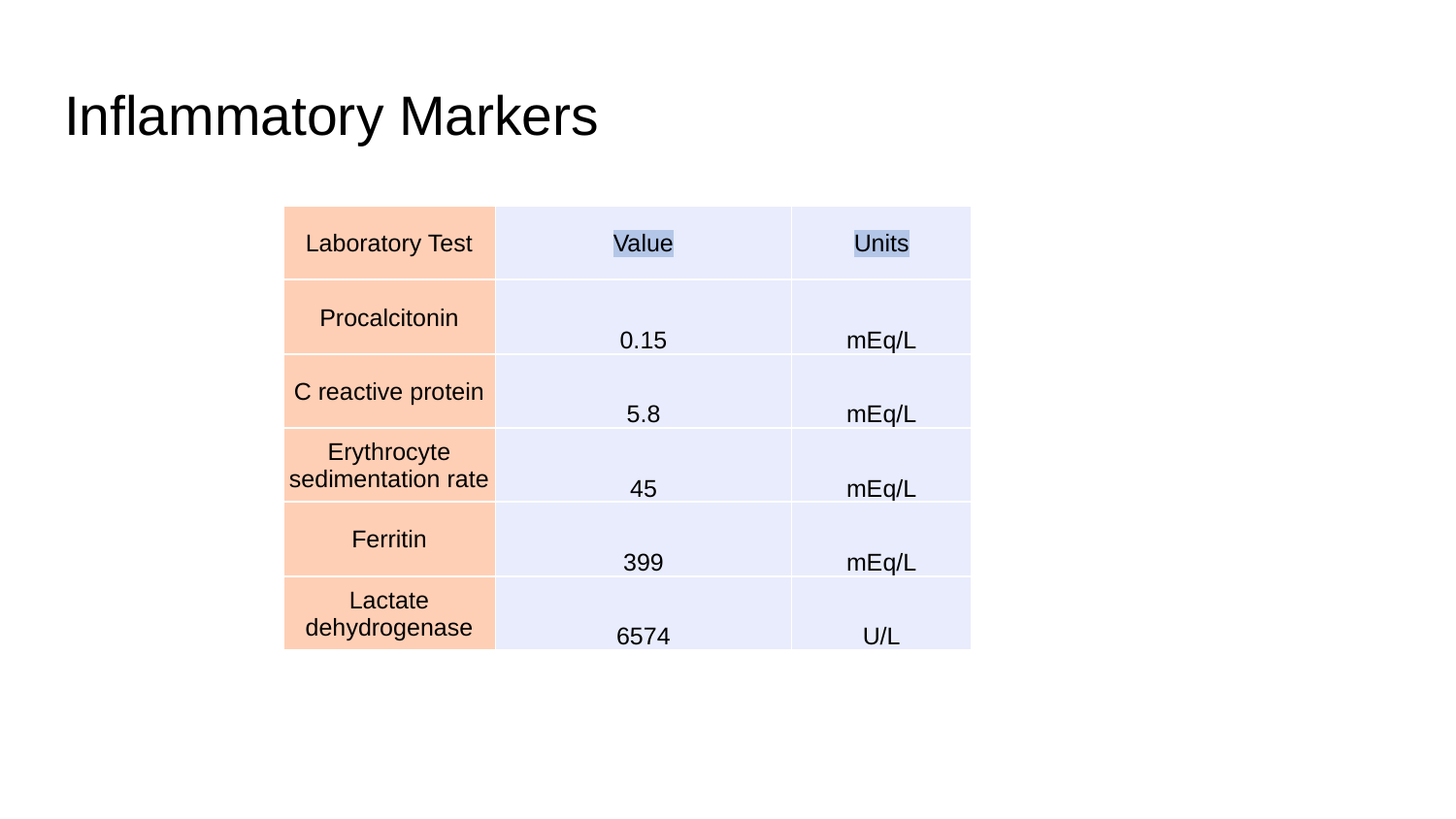

# Inflammatory Markers
| Laboratory Test | Value | Units |
| --- | --- | --- |
| Procalcitonin | 0.15 | mEq/L |
| C reactive protein | 5.8 | mEq/L |
| Erythrocyte sedimentation rate | 45 | mEq/L |
| Ferritin | 399 | mEq/L |
| Lactate dehydrogenase | 6574 | U/L |

## Slide 16
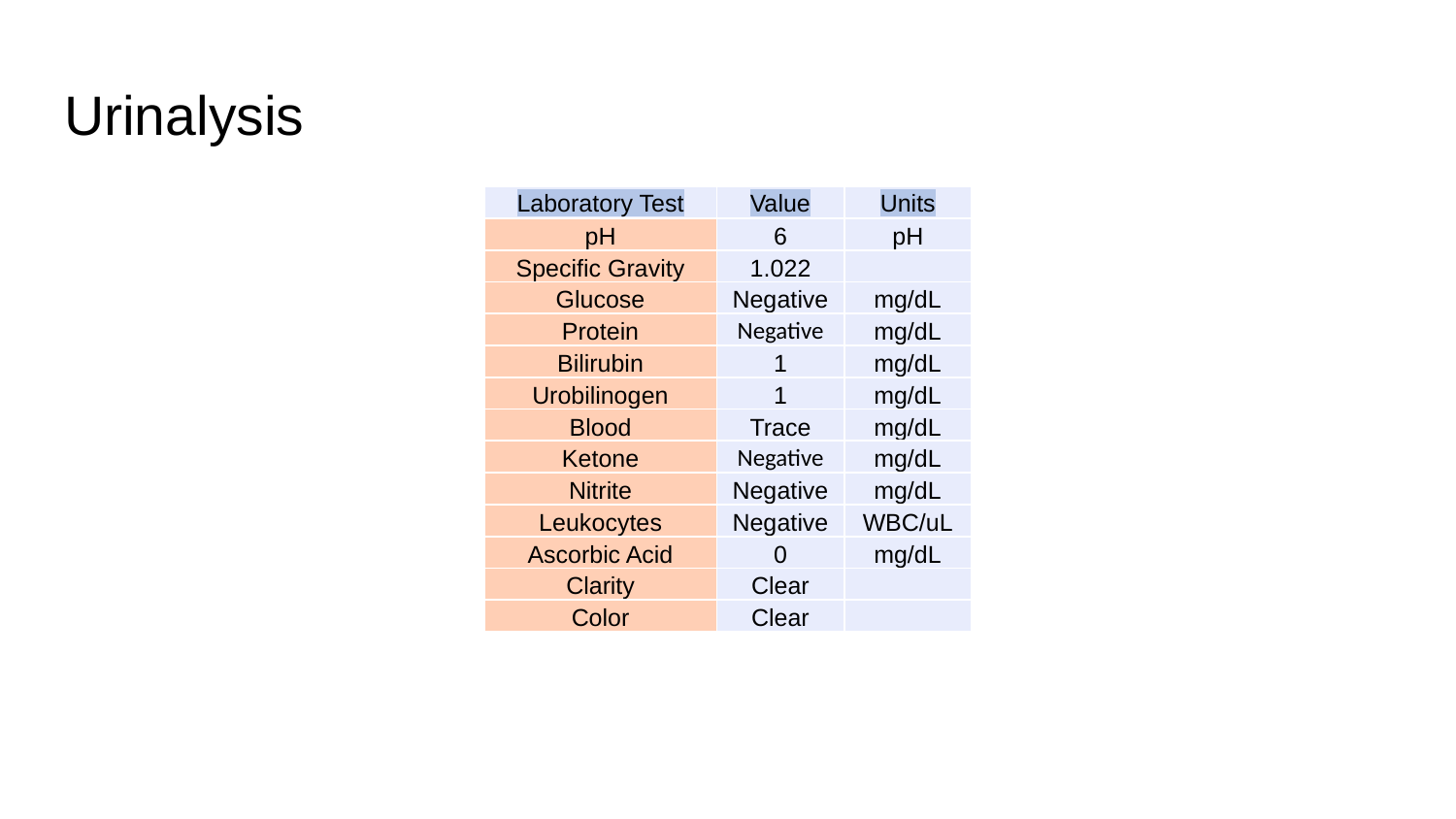

# Urinalysis
| Laboratory Test | Value | Units |
| --- | --- | --- |
| pH | 6 | pH |
| Specific Gravity | 1.022 | |
| Glucose | Negative | mg/dL |
| Protein | Negative | mg/dL |
| Bilirubin | 1 | mg/dL |
| Urobilinogen | 1 | mg/dL |
| Blood | Trace | mg/dL |
| Ketone | Negative | mg/dL |
| Nitrite | Negative | mg/dL |
| Leukocytes | Negative | WBC/uL |
| Ascorbic Acid | 0 | mg/dL |
| Clarity | Clear | |
| Color | Clear | |

## Slide 17
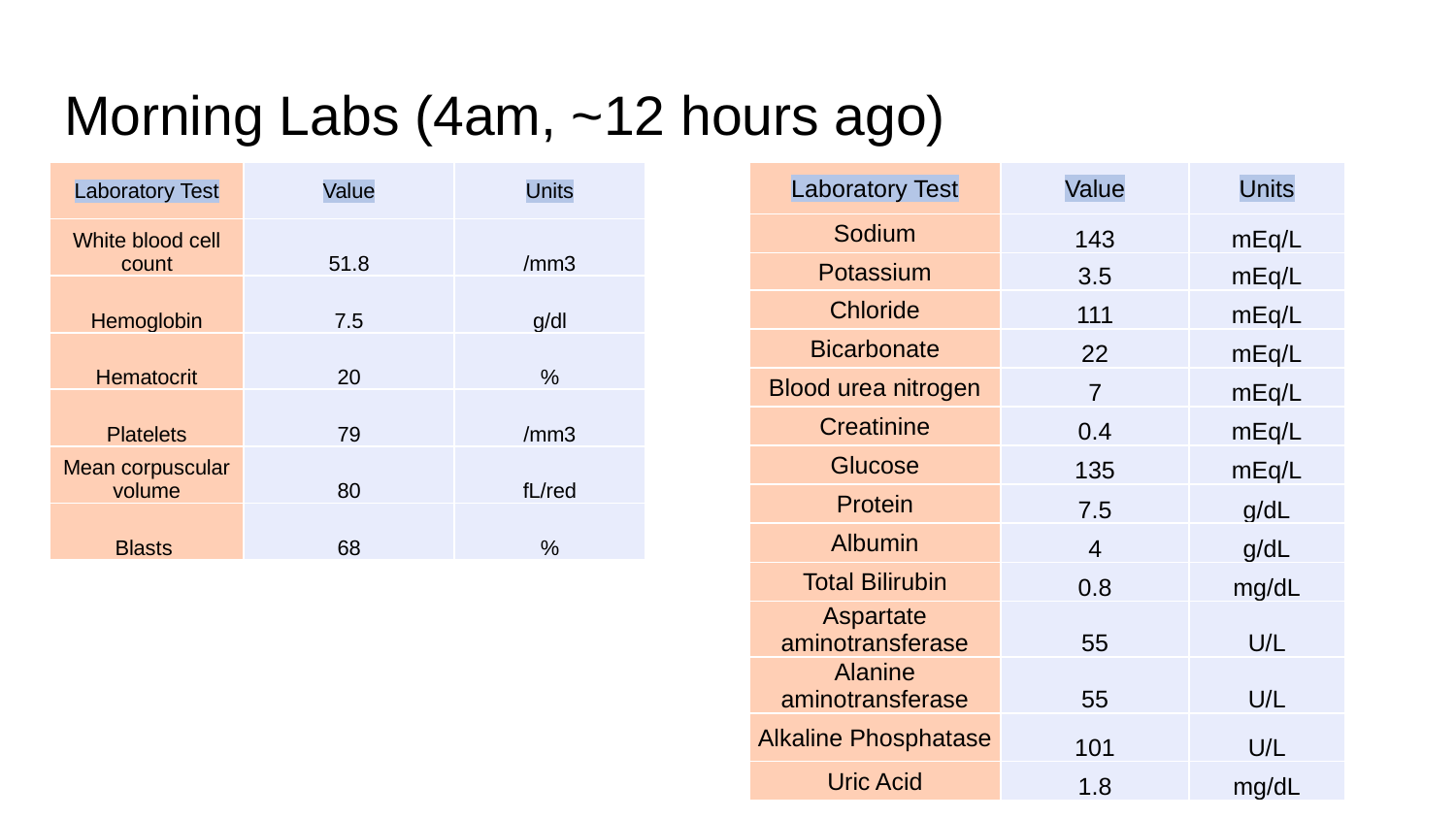

# Morning Labs (4am, ~12 hours ago)
| Laboratory Test | Value | Units |
| --- | --- | --- |
| White blood cell count | 51.8 | /mm3 |
| Hemoglobin | 7.5 | g/dl |
| Hematocrit | 20 | % |
| Platelets | 79 | /mm3 |
| Mean corpuscular volume | 80 | fL/red |
| Blasts | 68 | % |
| Laboratory Test | Value | Units |
| --- | --- | --- |
| Sodium | 143 | mEq/L |
| Potassium | 3.5 | mEq/L |
| Chloride | 111 | mEq/L |
| Bicarbonate | 22 | mEq/L |
| Blood urea nitrogen | 7 | mEq/L |
| Creatinine | 0.4 | mEq/L |
| Glucose | 135 | mEq/L |
| Protein | 7.5 | g/dL |
| Albumin | 4 | g/dL |
| Total Bilirubin | 0.8 | mg/dL |
| Aspartate aminotransferase | 55 | U/L |
| Alanine aminotransferase | 55 | U/L |
| Alkaline Phosphatase | 101 | U/L |
| Uric Acid | 1.8 | mg/dL |

## Slide 18
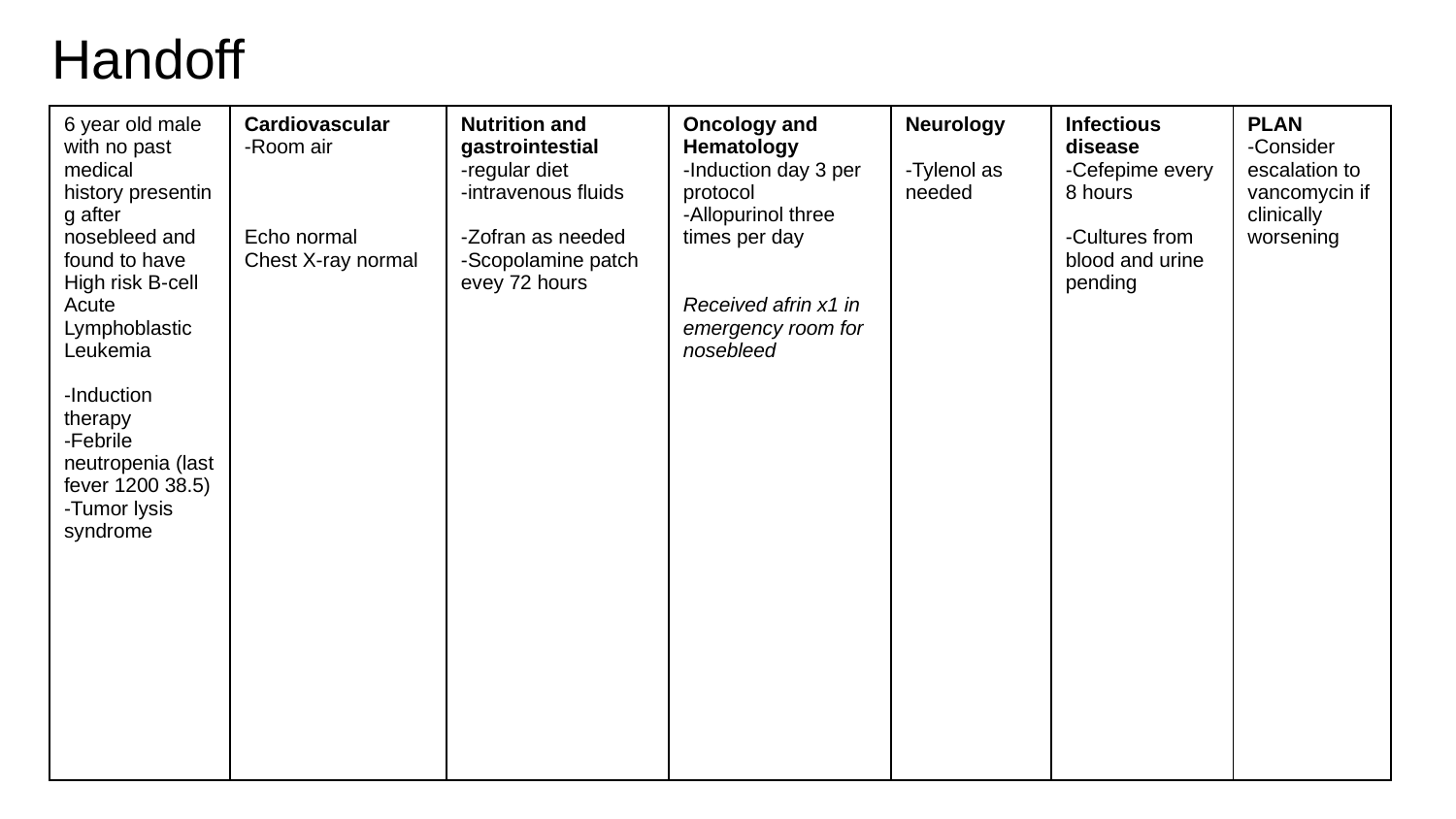

# Handoff
| 6 year old male with no past medical history presenting after nosebleed and found to have High risk B-cell Acute Lymphoblastic Leukemia -Induction therapy -Febrile neutropenia (last fever 1200 38.5) -Tumor lysis syndrome | Cardiovascular -Room air Echo normal Chest X-ray normal | Nutrition and gastrointestial -regular diet -intravenous fluids -Zofran as needed -Scopolamine patch evey 72 hours | Oncology and Hematology -Induction day 3 per protocol -Allopurinol three times per day Received afrin x1 in emergency room for nosebleed | Neurology -Tylenol as needed | Infectious disease -Cefepime every 8 hours -Cultures from blood and urine pending | PLAN -Consider escalation to vancomycin if clinically worsening |
| --- | --- | --- | --- | --- | --- | --- |
